# Protein neddylation and its role in health and diseases

**DOI:** 10.1038/s41392-024-01800-9

**Published:** 2024-04-05

**Authors:** Shizhen Zhang, Qing Yu, Zhijian Li, Yongchao Zhao, Yi Sun

**Affiliations:** 1 Department of Breast Surgery, the Second Affiliated Hospital, Zhejiang University School of Medicine, Hangzhou, 310029 China; 2 Cancer Institute (Key Laboratory of Cancer Prevention and Intervention, China National Ministry of Education), the Second Affiliated Hospital, Zhejiang University School of Medicine, Hangzhou, 310029 China; 3 Institute of Translational Medicine, Zhejiang University School of Medicine, Hangzhou, 310029 China; 4 Department of Thyroid Surgery, Zhejiang Cancer Hospital, Institute of Basic Medicine and Cancer (IBMC), Chinese Academy of Sciences, Hangzhou, 310022 China; 5 Key Laboratory of Head & Neck Cancer Translational Research of Zhejiang Province, Hangzhou, 310022 China; 6 Department of Hepatobiliary and Pancreatic Surgery, Zhejiang University School of Medicine, Hangzhou, 310029 China; 7 Zhejiang Provincial Key Laboratory of Pancreatic Disease, The First Affiliated Hospital, Zhejiang University School of Medicine, Hangzhou, 310029 China; 8 Zhejiang University Cancer Center, Hangzhou, 310029 China; 9 Leading Innovative and Entrepreneur Team Introduction Program of Zhejiang, Hangzhou, 310024 China; 10 Research Center for Life Science and Human Health, Binjiang Institute of Zhejiang University, Hangzhou, 310053 China

**Keywords:** Drug development, Cancer

## Abstract

NEDD8 (Neural precursor cell expressed developmentally downregulated protein 8) is an ubiquitin-like protein that is covalently attached to a lysine residue of a protein substrate through a process known as neddylation, catalyzed by the enzyme cascade, namely NEDD8 activating enzyme (E1), NEDD8 conjugating enzyme (E2), and NEDD8 ligase (E3). The substrates of neddylation are categorized into cullins and non-cullin proteins. Neddylation of cullins activates CRLs (cullin RING ligases), the largest family of E3 ligases, whereas neddylation of non-cullin substrates alters their stability and activity, as well as subcellular localization. Significantly, the neddylation pathway and/or many neddylation substrates are abnormally activated or over-expressed in various human diseases, such as metabolic disorders, liver dysfunction, neurodegenerative disorders, and cancers, among others. Thus, targeting neddylation becomes an attractive strategy for the treatment of these diseases. In this review, we first provide a general introduction on the neddylation cascade, its biochemical process and regulation, and the crystal structures of neddylation enzymes in complex with cullin substrates; then discuss how neddylation governs various key biological processes via the modification of cullins and non-cullin substrates. We further review the literature data on dysregulated neddylation in several human diseases, particularly cancer, followed by an outline of current efforts in the discovery of small molecule inhibitors of neddylation as a promising therapeutic approach. Finally, few perspectives were proposed for extensive future investigations.

## Introduction

**Fig. 1:**
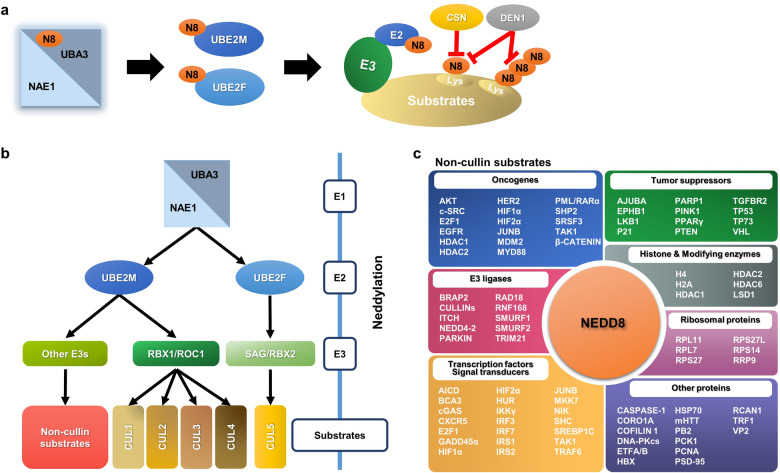
The neddylation pathway. **a** Three enzymes catalyze the NEDD8 attachment to a substrate: E1 activation enzyme, a heterodimer of catalytic subunit UBA3/NAEβ, and regulatory subunit NAE1/APPBP1; E2 conjugation enzymes, UBE2M/UBC12 and UBE2F; and E3 ligases (e.g., RBX1 and RBX2). One type of enzyme that removes NEDD8 from a substrate: Deneddylase (e.g CNS5). **b** Neddylation reaction with the UBE2M-RBX1 pair to catalyze neddylation of CULs 1-4, and UBE2M-other E3s pairs for neddylation of non-cullin substrates, and the UBE2F-RBX2 pair to catalyze CUL5 neddylation. **c** Classification of known non-cullin substrates

The protein post-translational modifications (PDM), such as phosphorylation, acetylation, methylation, glycosylation, nitrosylation, lipidation, ubiquitylation, and neddylation among others, affect many aspects of normal cell biology, and are essential for the maintenance of the homeostasis of a cell.^1,2^ Specifically, neddylation is an ubiquitylation-like (UBL) modifications.^3^ NEDD8, one of the UBLs with 59% sequence identity to ubiquitin,^4^ was synthesized as an 81 amino-acid precursor protein, then converted to the mature form by proteolytic cleavage to expose the C-terminal Gly 76.^5,6^ The neddylation leads to attachment of NEDD8 to the lysine reside of a substrate protein, catalyzed by a three-step enzymatic cascade of E1 NEDD8-activating enzyme (NAE), NEDD8-conjuagating enzyme E2 and substrate-specific NEDD8-E3 ligases.^7,8^ Like ubiquitin, the NEDD8 is first adenylated and activated by NAE in the presence of ATP,^9^ and activated NEDD8 is then transferred to UBE2M (also known as UBC12) or UBE2F, two known E2s via a trans-thiolation reaction.^10,11^ Finally, an NEDD8 E3 ligase, such as RING-box proteins (RBX1/2), catalyzes the transfer of NEDD8 from E2 to its substrate protein via a covalent bond^12^ (Fig. 1). Unlike ubiquitylation that normally conjugates ubiquitin to substrate through poly-ubiquitin chains mainly for subsequent degradation, NEDD8 is mainly conjugated to a single lysine residue on substrates leading to mono-neddylation.^13^ Interestingly, recent proteomics studies provided direct evidence for the existence of poly-neddylation, and the NEDD8 chains are also capable of being linked on K6, K11, K22, K27, K48 and K54 resides on NEDD8.^14–16^ The substrate proteins, upon neddylation modification, are not doomed for degradation, rather have altered properties, such as the changes in structural conformation, stability, activity/binding affinity, or subcellular localization, eventually leading to altered substrate functions.^8^

In mammalian cells, neddylation substrates are mainly classified into two categories: Cullin family members and non-cullin proteins^17^ (Fig. 1, Table 1). The cullin family has 8 members, including cullin-1, cullin-2, cullin-3, cullin-4A, cullin-4B, cullin-5, cullin-7, and cullin-9 with last two members less studied. Cullins are the best characterized physiological substrates of neddylation and their neddylation modification causes the conformational change to disrupt inhibitory binding of CAND1 (Cullin-associated and neddylation-dissociated 1), leading to activation of Cullin RING ligases (CRLs). By activating the CRLs, neddylation pathway controls the ubiquitylation of ~20% of cellular proteins doomed degradation via the ubiquitin-proteasome system,^18^ thus regulating various cellular processes and homeostasis.^12,19–22^

**Table 1:** Neddylation substrates, their catalyzing enzymes and biochemical outcomes

Neddylation substrates	Neddylation enzymes				Biological function	Related biological processes or diseases	Citation references
	E1	E2	E3	Deneddylase			
AICD (APP intracellular domain)	NAE	UBE2M	Unknown	Unknown	Inhibiting its transcription activity	Unknown	^499^
Ajuba (JUB)	NAE	Unknown	Hakai	Unknown	Reducing its stability by promoting its degradation.	Hepatocellular carcinoma	^500^
Akt	NAE	Unknown	Unknown	DEN1	Increasing its stability	Hepatocellular carcinoma	^333^
BCA3 (AKIP1)	NAE	Unknown	Unknown	DEN1	Inhibiting NF-κB transcription	Breast cancer	^501^
BRAP2 (BRAP)	NAE	Unknown	Unknown	Unknown	Promoting TNF-α-induced NF-κB translocation	Inflammatory responses	^502^
Caspase-1	NAE	UBE2M	Unknown	Unknown	Promoting cleavage of CARD from endogenous pro-caspase-1 upon inflammasome activation.	Inflammatory responses	^503^
cGAS	NAE	UBE2M	RNF111	DEN1	Essential for DNA-triggered cGAS activation.	Antiviral defense	^177^
Cofilin 1 (CFL1)	NAE	Unknown	Unknown	DEN1	Regulation of neuronal actin organization	Neuronal development	^14^
Coro1a	NAE	UBE2F	TRIM40	Unknown	Promoting Rab7 recruitment to multivesicular bodies and regulating production of tumor extracellular vesicles (EVs).	Cancer	^504^
c-Src	NAE	Unknown	CBL, CBLB	DEN1	Inducing its ubiquitylation and degradation	Cancer	^505^
Cullins	NAE	UBE2M/UBE2F	RBX1/SAG	CSN, DEN1	Activating cullin-RING ligases	Cancer, cardiovascular diseases, autoimmune diseases, et al.	^506,507^
CXCR5	NAE	Unknown	Unknown	Unknown	Targeting CXCR5 to the cell membrane, stimulating cell migration and motility.	Unknown	^508^
DNA-PKcs	NAE	UBE2M	HUWEI	Unknown	Promoting the signaling cascades in theNHEJ repair following DNA damage	Cancer	^151,152^
E2F1	NAE	UBE2F	Unknown	DEN1	Reducing its stability and transcriptional activity	Cancer	^150,509^
EGFR	NAE	Unknown	CBL	CSN	Enhancing its ubiquitylation and subsequent lysosomal degradation	Cancer	^129,510^
EphB1	NAE	Unknown	Unknown	Unknown	Increasing its kinase activity by preventing its degradation	Liver Fibrosis	^286^
ETFA/B	NAE	Unknown	Unknown	Unknown	Inhibiting its ubiquitylation and subsequent degradation	Fasting-induced liver steatosis	^238^
Gadd45a	NAE	Unknown	Unknown	DEN1	Promoting its nuclear export	Unknown	^511^
H2A	NAE	UBE2M	RNF168	DEN1	Antagonizing its ubiquitylation; Nedd8 inhibits the DNA damage repair process via suppressing the ubiquitylation of H2A and γH2AX, which further blocks the recruitment of BRCA1, a damage response protein.	DNA damage repair	^154^
HBx	NAE	UBE2M	HDM2	DEN1	Increasing its stability by inhibiting its ubiquitination	HBV-driven tumor growth	^278^
HDAC1	NAE	Unknown	Unknown	DEN1	Facilitating its ubiquitination and decreasing its expression.	Acute myelogenous leukemia	^142^
HDAC2	NAE	Unknown	Unknown	DEN1	Promoting its proteasomal degradation	Atherogenesis	^143^
HDAC6	NAE	Unknown	Unknown	Unknown	Stimulating its deacetylase activity	Atherogenesis	^145^
HER2 (ERBB2)	NAE	Unknown	Unknown	Unknown	Inhibiting its ubiquitylation and subsequent degradation	Breast cancer	^131^
HIF1a	NAE	Unknown	Unknown	CSN	Promoting its stability	Cancer	^512^
HIF2a (EPAS1)	NAE	Unknown	Unknown	Unknown	Promoting its stability	Cancer	^512^
Histone H4	NAE	UBE2M	RNF111	Unknown	Activating DNA damage-induced ubiquitination	DNA damage repair	^44^
HSP70 (HSPA)	NAE	UBE2M	Unknown	DEN1	Mono-NEDD8 activates the ATPase activity of HSP70, whereas poly-neddylation inhibits HSP70 activity and apoptosis induction upon DNA damage	Hepatocellular carcinoma	^61^
mHTT	NAE	Unknown	Unknown	Unknown	Facilitating aggrephagy of its aggregates and presenting a signal for autophagic degradation.	Proteotoxic stress	^513^
HuR (ELAVL1)	NAE	Unknown	MDM2	DEN1	Increasing its stability and promoting its nuclear localization	Liver and colon cancer	^330^
IKKγ (IKBKG)	NAE	Unknown	TRIM40	Unknown	Inhibiting NF-κB signaling	Gastrointestinal cancers	^45^
IRF3	NAE	UBE2M	Unknown	Unknown	Promoting its nuclear translocation and transcriptional activity	Host anti-viral innate immunity	^514^
IRF7	NAE	UBE2M	Unknown	Unknown	Promoting its nuclear translocation and transcriptional activity	Host anti-viral innate immunity	^514^
IRS1/2	NAE	Unknown	C-CBL	DEN1	Promoting its ubiquitination-dependent degradation	Cancer cell migration	^515^
Itch	NAE	Unknown	Unknown	Unknown	Promoting the degradation of FoxO1 in regulating Tfh cell differentiation	Infectious diseases	^200,205^
JunB	NAE	Unknown	Itch	Unknown	Reducing its transcriptional activity and stability	Unknown	^516^
LKB1 (STK11)	NAE	Unknown	Unknown	Unknown	Increasing its stability	Hepatocellular carcinoma	^333^
LSD1 (KDM1A)	NAE	UBE2M	Unknown	Unknown	Promoting its ubiquitination-dependent degradation	Gastric cancer	^517^
MDM2	NAE	Unknown	MDM2	DEN1	Promoting MDM2 protein stabilization	Unknown	^43,148^
MKK7 (MAP2K7)	NAE	UBE2M	RanBP2	Unknown	Decreasing its basal kinase activity	Breast cancer	^328^
MYD88	NAE	UBE2M	Unknown	DEN1	Antagonizing its ubiquitination and suppressing MyD88-dependent NF-κB signaling.	Innate immunity response	^518^
NEDD4-2	NAE	UbcH12	Unknown	DEN1	Enhanced ubiquitin E3 ligase activity	Unknown	^519,520^
NIK (MAP3K14)	NAE	UBE2M	Unknown	DEN1	Promoting its proteasomal degradation.	Acute liver failure.	^115^
P21 (CDKN1A)	NAE	Unknown	cIAP1	Unknown	Promoting its proteasomal degradation in lieu of ubiquitination	Cancer	^521^
Parkin (PRKN)	NAE	UBE2M	CBL	DEN1	Increasing its E3 ligase activity	Parkinsonism	^297,298^
PARP1	NAE	UBE2M	Unknown	DEN1	Attenuating PARP‐1 activation after oxidative stress and delaying the initiation of PARP‐1‐dependent cell death	DNA damage response	^62^
PB2	NAE	Unknown	HDM2	Unknown	Reducing its stability.	Influenza A virus infection	^312^
PCK1	NAE	Unknown	Unknown	Unknown	Promoting its enzyme activity	Glucose metabolism	^244^
PCNA	NAE	UBE2M	RAD18	DEN1	Impairing PCNA monoubiquitination	DNA damage response	^522^
PINK1	NAE	UBE2M	Unknown	DEN1	Stabilizing PINK1 55 kDa fragment	Parkinsonism	^297^
PML/RARα	NAE	UBE2M	Unknown	DEN1	Increasing its DNA-binding activity and further inhibits the phase separation of the PML moiety, leading to disruption of PML NB construction	Acute promyelocytic leukemia	^473^
PPARγ (PPARG)	NAE	Unknown	MDM2	DEN1	Increasing its stability by competing with ubiquitination	Obesity	^251^
PSD-95 (DLG4)	NAE	UBE2M	MDM2	DEN1	Promoting its clustering function	Spine development	^165^
PTEN	NAE	UBE2M	XIAP	DEN1	Promoting its nuclear import and enhancing the activity of PI3K/Akt signaling	Cancer	^256^
RAD18	NAE	Unknown	RAD18 (autoneddylation)	DEN1	Regulating its localization and antagonizing its ubiquitination	DNA damage response	^523^
RCAN1	NAE	UbcH12	Unknown	Unknown	Blocking its ubiquitylation and degradation; increasing its binding to calcineurin	Unknown	^479^
RNF168	NAE	UBE2M	RNF168(autoneddylation)	DEN1	Activation of its E3 ligase activity	DNA damage repair	^154^
RPL11	NAE	Unknown	MDM2	DEN1	Promoting its stabilization and nucleolar localization	Nucleolar stress response	^157–159^
RPL7	UBE1	Unknown	HUWE1	Unknown	Promoting its stabilization and nucleolar localization	Proteotoxicity stress response	^524^
RPS14	NAE	Unknown	MDM2	DEN1	Increasing its stability and correct localization	Cancer	^160^
RPS27/ RPS27L	NAE	Unknown	MDM2	DEN1	Increasing their stability	Cancer	^161^
RRP9	NAE	Unknown	SMURF1	DEN1	Promoting pre-rRNA processing to promote cell proliferation.	Cancer	^329^
Shc	NAE	UBE2M	Unknown	Unknown	Facilitating the formation of a ZAP70-Shc-Grb2 axis to regulate the downstream Erk activation	T-cell function	^132^
SHP2 (PTPN11)	NAE	UBE2M	XIAP	DEN1	Impairing its activation.	Colon cancer	^331^
SMURF1	NAE	UBE2M	SMURF1	DEN1	Enhancing its E3 ubiquitin ligase activity.	Cancer	^46^
SMURF2	NAE	UBE2M	Unknown	DEN1	Enhancing the ubiquitin ligase activity of Smurf2 and promoting its ubiquitylation and degradation.	Unknown	^525^
SREBP1C	NAE	Unknown	HDM2	DEN1	Competing with its ubiquitylation to stabilize SREBP1c to promote hepatic steatosis	Nonalcoholic fatty liver	^255^
SRSF3	NAE	Unknown	Unknown	Unknown	Reducing its stability by promoting its degradation.	Hepatocellular carcinoma	^283^
TAK1 (MAP3K7)	NAE	Unknown	Unknown	DEN1	Promoting its nuclear import	Unknown	^511^
TGFBR2	NAE	UBE2M	c-CBL	DEN1	Antagonizing the ubiquitination and degradation of the TβRII	Leukemia	^47^
TP53	NAE	UBE2M	MDM2, SCF^Fbxo11^	DEN1	Triggering cytoplasmic localization to inhibit its transcriptional activity	Cancer	^43,162,526^
TP73	NAE	Unknown	MDM2	DEN1	Triggering cytoplasmic localization to inhibit its transcriptional activity	Unknown	^527^
TRAF6	NAE	Unknown	Unknown	Unknown	Being essential for IL-17A-induced NF-κB activation	Inflammatory arthritis	^528^
TRF1 (TERF1)	NAE	UBE2M	FBX4	Unknown	Promoting its proteasomal degradation in a NUB1-dependent manner	Telomere maintenance	^529^
TRIM21 (RNF81)	NAE	UBE2M	Unknown	Unknown	Promoting its E3 ubiquitin ligase	Obesity-induced inflammation and metabolic disorders	^187^
VHL	NAE	UBE2M	MDM2	Unknown	Precluding ECV (Elongin B/C–CUL2–VHL) complex formation and promoting fibronectin extracellular matrix assembly.; preventing its binding with p53	Cancer	^123,124^
VP2	NAE	Unknown	XIAP	Unknown	Reducing its stability by promoting its degradation	Restrict enterovirus 71 (EV71) replication	^313^
β-catenin (CTNNB1)	NAE	Unknown	β-TrCP2	DEN1	Promoting its degradation to inhibit β-catenin signal	Cortical development	^164,530^

The non-cullin substrates can be further classified into seven sub-groups, including oncogenes, tumor suppressor, ribosomal proteins, histone and modifying enzymes, transcription factors and signal transducers, E3 ligases, and quite few others (Fig. 1c and Table 1). The neddylation modifications of these proteins further regulates the biological processes controlled by these substrates. However, most studies on non-cullin substrates involves the use of overexpression system, making it controversy as to whether they are indeed physiological substrates of neddylation. Furthermore, the proteomics-based approaches to identify new neddylation substrates faced major technical challenges due to low abundancy, transient/reversible nature, and contamination of ubiquitin or other ubiquitin-like proteins, among others.^15,23^

**Fig. 2:**
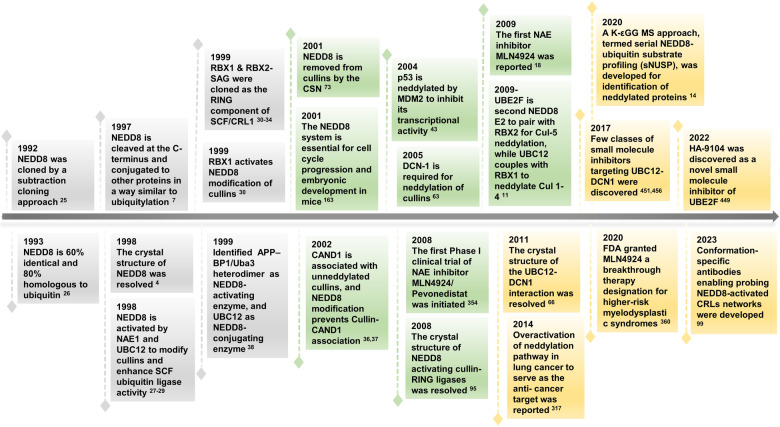
The chronicles of neddylation-related research. Timeline showing important discovery events in the field of neddylation research over past 30 years: From the cloning of NEDD8, demonstration of NEDD8 conjugates to cullins, identification of neddylation E1 and E2s, revelation of neddylation E2/E3 crystal structures, to discovery of neddylation E1 inhibitor (MLN4924/Pevonedistat) and its clinical trials, and its FDA approval for the treatment of MDS

It has been for more than 30 years, since the first discovery of NEDD8 in 1992. Figure 2 summarized the milestone discoveries in the neddylation field, covering from target identification, validation, drug discovery to clinical trials, and clinical approval of a neddylation inhibitor for treatment of human diseases. In this review, we first introduce the components of neddylation cascade, including their crystal structures with substrates, then summarize the roles of neddylation pathway in regulation of normal cell functions and its dysregulation in several human diseases, particularly cancer, if abnormally activated. We then provide an overview on the clinical significances of targeting neddylation pathway. Finally, we summarize the current drug discovery efforts for neddylation inhibitors, and end up with few proposed future perspectives.

## Biochemical process of neddylation and deneddylation

The neddylation cascade consists of a single E1 (NAE), two E2s (UBE2F and UBE2M, also known as UBC12), and a few E3s, catalyzing a reaction to add the NEDD8 to its substrates, including cullins or non-cullin proteins to add NEDD8 to a substrate (Fig. 1). The deneddylation is a reverse reaction to remove NEDD8 from a neddylated substrate, catalyzed by an NEDD8 isopeptidase, such as COP9 signalosome complex (CSN).^24^ Physiologically, both neddylation and deneddylation processes are essential for the viability of most organisms,^8^ and deregulated neddylation or deneddylation is involved in the development of several human diseases, including neurodegenerative disorders and cancers.^12,21^

### NEDD8

NEDD8 was first cloned in 1992 as a highly expressed gene in mouse neural precursor cells,^25^ and characterized in 1993 to encode a small new peptide with 81 amino acids, sharing 59% identical and 80% homologous to ubiquitin, and conservatively expressed in most eukaryote and almost all tissues.^7,26^ It was further defined in 1997 that newly synthesized NEDD8 with 81 amino-acid is actually a precursor, which is cleaved by C-terminal hydrolases, including UCH-L3 and NEDP1/DEN1/SENP8 into mature form of 76 amino acids with glycine 76 at its C-terminus. The mature NEDD8 is then conjugated to other proteins similar to ubiquitylation.^7^ In 1998, the crystal structure of NEDD8 was resolved with 3D structure similar to ubiquitin.^4^ Also in 1998, a new NEDD8-ligating system was established for cullin-4A, involving NEDD8 activation by NAE E1 and conjugation by UBC12 E2, eventually the attachment of NEDD8 to cullin-4A.^27^ And also RUB1, a yeast NEDD8, was identified to conjugate to Cullin-1/CDC53 when coupling with ULA1 and UBA3, a heterodimeric E1, and UBC12 E2 to modulate ubiquitin-proteasome system.^27–29^ While two neddylation E3s RBX1 (also known as ROC1 or Hrt1) and RBX2/SAG were cloned in 1999,^30–33^ SAG was first cloned as an antioxidant protein,^34^ then characterized as RBX1 family member with ligase activity in 2000.^35^ In 2002, it was found that NEDD8 not only enhances intrinsic ubiquitylation activity of CRLs upon attachment to cullins, but also prevents cullin binding with its inhibitor CAND1 to maintain fully functional CRLs.^36,37^

### NEDD8-activating enzyme E1 (NAE)

NAE consists of catalytic subunit UBA3 (also known as NAEβ) and the regulatory subunit amyloid-beta precursor protein-binding protein 1 (APPBP1, also known as NAE1).^38^ NAE activates NEDD8 to initiate neddylation process by recognizing the alanine 72 in NEDD8,^4,27^ and continues the NEDD8 transfer cascade by activating the C-terminus of NEDD8 with diGly.^9^ There are two distinct active sites resided in NAE, an adenylation site located in UBA3 and a cysteine transthiolation domain located in residues involved in both subunits.^9^ The holo-enzyme of NAE is an “open” conformation when a large groove separates these two active sites. The third functional domain is the C-terminal ubiquitin fold domain (UFD) in UBA3, which is important for binding to NEDD8 E2 enzymes.^9^ The catalytic reaction by NAE is highly ordered, involving the conversion of ATP to AMP, and “open” to “close” conformations.^39,40^ Specifically, NEDD8 is linked to the active site cysteine of NAE via its C-terminus in the “closed” state, whereas the next NEDD8 binding to the adenylation site again will re-adopt the NAE to the “open” conformation.^41^

### NEDD8-specific E2 conjugating enzymes

Mammalian cells have two NEDD8-specific E2 conjugating enzymes, namely, UBE2F and UBE2M (also known as UBC12), which catalyze the transthiolation reaction to transfer NEDD8 from the active cysteine site of NAE onto the active cysteine site on an E2 enzyme.^10,11^ The C-terminal UFD of UBA3 is responsible for E2 enzyme interaction.^10^ Specifically, when NAE is double-NEDD8 loaded, the UFD starts to reorient and form a cryptic E2-binding groove together with a portion of the adenylation domain.^41^ Afterwards, E2 directly binds to the moiety of NEDD8 attached to the NAE.^41^ In addition, the N-terminus of E2 binding in a groove of the adenylation domain of UBA3 appears to be the third E1-E2 interface, which is unique to the NEDD8 cascade.^42^ These binding interfaces create an amicable environment for catalysis by positioning the cysteine residue of NAE and E2 enzyme in the close proximity. After transthiolation, two E2 binding sites with doubly NEDD8-loaded NAE are lost, and thus the E1-E2 binding becomes weak, followed by E2 release from E1.^17^

### NEDD8 E3 ligases

More than a dozen of neddylation E3 ligases were identified, including RBX1/2,^11,17,30^ murine double minute 2 (MDM2),^43^ ring fingers protein 111 (RNF111),^44^ tripartite motif (TRIM) 40,^45^ Smurf1,^46^ casitas B-lineage lymphoma (c-CBL)^47^ and yeast Tfb3 (MAT1 in mammal)^48^ among others^12^ (Table 2). All these E3s are dual E3s for both neddylation and ubiquitylation which are capable of transferring either NEDD8 or ubiquitin from E2s onto the substrate proteins. RBX1 and RBX2/SAG are neddylation E3s for cullin neddylation. RBX1 couples with UBE2M E2 to promote neddylation of cullins 1-4 (CUL1-4), whereas RBX2/SAG couples with UBE2F E2 for cullin-5 (CUL5) neddylation.^11,49^ As the dual E3s, RBX1 is the RING component of CRLs 1–4, whereas RBX2/SAG is the RING component of CRL5, required for CRL activity (Fig. 1b). The RING domain of RBX1/2 is structurally organized to bind with two zinc atoms in a cross-braced arrangement, which forms a protein–protein interaction interface for various E2s.^50^ And such interaction allows the catalyzation and transfer of the NEDD8 onto a lysine residue of the substrate protein. Since the catalytic domain between UBE2M and ubiquitin-specific E2s has similar structure, it is likely that NEDD8 E2 would interact with E3 RING domain similarly as ubiquitin-specific E2s binding to E3s.^51–54^ In fact, UBE2M was reported to serve as an ubiquitin E2 for CRL3 E3 under the physiological condition, and Parkin E3 under stressed conditions.^55^ However, how E3 ligases for non-cullin substrates interact with neddylation E2 (UBE2M or UBE2F) has not been systematically studies which is an interesting area for future investigation.

**Table 2:** Neddylation E3 ligase, deneddylases and their substrates

Neddylation E3 ligases	Substrates and references
c-CBL	c-Src,^505^ EGFR,^129,510^ TGFBR2,^47^ Parkin,^297,298^ IRS1/2^515^
cIAP1	P21 (CDKN1A)^521^
FBX4(SCF)	TRF1 (TERF1)^529^
FBXO11(SCF)	TP53^162^
β-TrCP2(SCF)	β-catenin (CTNNB1)^164,530^
Hakai	Ajuba (JUB)^500^
HDM2/MDM2	HBx,^278^ PB2,^312^ SREBP1C,^255^ HuR (ELAVL1),^330^ MDM2,^43^ PPARγ (PPARG),^251^ PSD-95 (DLG4),^165^ RPL11,^157–159^ RPS14,^160^ RPS27/ RPS27L,^161^ TP53,^43^ TP73,^527^ VHL^123,124^
HUWE1	DNA-PKcs,^151,152^ RPL7^524^
Itch	JunB^516^
RAD18	PCNA,^522^ RAD18^523^
RanBP2	MKK7 (MAP2K7)^328^
RBX1/2	Cullins^506^
RNF111	cGAS,^177^ Histone H4,^44^
RNF168	H2A,^154^ RNF168^154^
SMURF1	RRP9,^329^ SMURF1^46^
TRIM40	Coro1a,^504^ IKKγ (IKBKG)^45^
Tfb3	yeast Cul4-type cullin Rtt101^48^
XIAP	PTEN,^256^ SHP2 (PTPN11),^331^ VP2^313^
Deneddylases	
CSN	Cullins,^531^ EGFR,^129,510^ HIF1α^512^
SENP8/NEDP1/DEN1	Akt,^333^ BCA3 (AKIP1),^501^ cGAS,^177^ Cofilin 1 (CFL1),^14^ culins,^507^ c-Src,^505^ E2F1,^150,509^ Gadd45a,^511^ H2A,^154^ HBx,^278^ HDAC1,^142^ HDAC2,^143^ HSP70(HSPA),^61^ HuR (ELAVL1),^330^ IRS1/2,^515^ MDM2,^43,148^ MYD88,^518^ NEDD4-2,^519,520^ NIK (MAP3K14),^115^ Parkin (PRKN),^297,298^ PARP1,^62^ PCNA,^522^ PINK1,^297^ PML/RARα,^473^ PPARγ (PPARG),^251^ PSD-95 (DLG4),^165^ PTEN,^256^ RAD18,^523^ RNF168,^154^ RPL11,^157–159^ RPS14,^160^ RPS27/RPS27L,^161^ RRP9,^329^ SHP2 (PTPN11),^331^ SMURF1,^46^ SMURF2,^525^ SREBP1C,^255^ TAK1 (MAP3K7),^511^ TP53,^43^ TP73,^527^ β-catenin (CTNNB1)^164,530^
Ataxin-3	Unknown^532^
USP21	Unknown^533^
UCH-L1	Unknown^534^
UCH-L3	Unknown^535^
PfUCH54	Unknown^536^

### Neddylation chains formation and its regulation

In the most cases, NEDD8 is conjugated to a single Lys residue of a substrate to form mono-neddylation.^13^ However, in vitro, NEDD8 is capable of forming multiple NEDD8 chains on their substrates by the linkage via Lys6, Lys11, Lys22, Lys27, Lys48, Lys54, and Lys60.^14,15,56–58^ The poly-NEDD8 chains could be pre-formed on the catalytic cysteine residue of E2, then transferred to substrates.^59^ Recent proteomics studies also confirmed the existence of polyneddylated chains.^14–16,60^ However, for in vivo substrates, the neddylation occurs mainly in mono-NEDD8 form on a single or few conserved lysine residues, and the physiological significance of poly-NEDD8 chains remains elusive.^58^ Two recent studies showed possible role of poly-neddylated form. The first study reported that the conversion from poly-NEDD8 chains to mono-NEDD8 could act as a regulatory module for the function of HSP70 (heat shock protein 70), which is required for apoptosis induction upon DNA damage and may also be involved in tumorigenesis.^61^ The second study showed that the PARP-1 [poly(ADP-ribose) polymerase 1] can be poly-neddylated (tri-NEDD8) upon oxidative stress, and neddylated PARP-1 attenuates its activation to protect cells from death induced by the stress.^62^ Nevertheless, more extensive studies are needed to precisely characterize the physiological or pathological effect of substrate polyneddylation.

Few mechanisms have been elucidated as to how the neddylation process is being regulated. The yeast defective in cullin neddylation 1 (DCN1) protein, which bind to both NEDD8 E2 enzymes and cullins to increase neddylation efficiency, is considered as the main regulator of neddylation.^63–65^ DCN1 appears to constrain the RBX1–UBE2M ~ NEDD8 complex structurally to direct NEDD8 towards a most optimal way by recognizing the site specifically in the cullin close to its neddylation site and the acetylated N-terminus of the UBE2M.^65,66^ In budding yeast DCN1 is required for neddylation of CUL1 and CUL3, but not CUL4,^48^ suggesting that other regulators are also involved in regulating different cullins. Human cells have 5 DCN1-like proteins (DCNL), namely DCNL1–DCNL5. They all share a conserved C-terminal potentiating neddylation (PONY) domain, which is necessary and sufficient for cullin neddylation.^64^ DCNL1 and DCNL2 are the yeast DCN1 homologs, which interact with cullins and mainly modulate neddylation of CUL1 and CUL3.^67^ DCNL3 is mainly located at the plasma membrane where it also contributes to CUL3 neddylation,^67^ while DCNL4 and DCNL5 with a nuclear localization signal are exclusively localized in the nucleus with unknown cullin preference.^68^ Additionally, the neddylation process can be regulated by a protein synthesis enzyme glycyl-tRNA synthetase, which binds to NAE1 to facilitate capture and protection of activated E2 UBE2M before it reaching to a downstream substrate.^69^ More recently, we reported that neddylation is subject to self-regulation. Specifically, UBE2M could act as an ubiquitin E2. Under physiological condition, UBE2M couples with Cul-3, whereas under stressed conditions, including exposure to TPA (12-O-tetradecanoylphorbol-13-acetate) or hypoxia, UBE2M couples with Parkin E3 ligase to promote UBE2F ubiquitylation, therefore blocking CUL5 neddylation.^55^ Finally, in bacteria, NEDD8 can be directly targeted. Specifically, upon bacterial infection, the Cif (Cycle-inhibiting factor) from enteropathogenic Escherichia coli (EPEC) and its homolog from Burkholderia pseudomallei (CHBP) can convert the Gln40 residue to a Glu on NEDD8, therefore abrogating neddylation process.^70,71^

### Deneddylation

Deneddylation is a process by which NEDD8 is removed from a conjugated substrate, catalyzed by deneddylases. The isopeptidases that deconjugate NEDD8 moieties from substrates in vitro, include Constitutive photomorphogenesis 9 (COP9) signalosome (CSN), NEDP1/DEN1, Ataxin-3, USP21, UCH-L1, and UCH-L3^8,72,73^ (Table 2). However, only CSN and DEN1 are NEDD8 specific, the rest are also capable of deubiquitylation.^8^ The CSN is the major deneddylase comprising a metalloprotease active site that can cleave the NEDD8 from both the cullin and other non-cullin proteins.^74–76^ Given that genetic deletion of any subunits of CSN cause tissue damages or even lethality, CSN is critical and even indispensable for tissue homeostasis and development.^77–82^ CSN5, the catalytic subunit of CSN, is auto-inhibited in the form of substrate-free holoenzyme,^83^ Upon the interaction between the C-terminus of the cullin and the CSN subunits CSN2/CSN4, CSN5 is allosterically activated with association of CSN6, to form active deneddylase.^84,85^ Since the CSN tightly binds the deneddylated CRL, the RBX1 is unable to cooperate with activated NEDD8 E2 due to the steric hindrance, while the CRL substrates are capable of competing away CSN, leading to CRL activation by neddylation.^86,87^ Likewise, the cysteine protease DEN1/NEDP1 also specifically binds to NEDD8 and mainly deconjugate NEDD8 from non-cullin proteins,^88^ as well as cullin proteins.^89^ Therefore, DEN1 plays a complementary role in deneddylation to the CSN complex in vivo.^88^ Notably, DEN1 is more likely to prevent aberrant poly-neddylation via deneddylation, since it tends to deneddylate hyper-neddylated cullins without affecting mono-neddylation.^89^ Moreover, DEN1 appears to selectively remove the NEDD8 chains with K6-, K11- and K54-linkages, given that depletion of DEN1 causes the accumulation of these linkages of NEDD8 chains.^14^

## Crystal structures of neddylation enzymes in complex with cullin substrates

Cullin neddylation is a complex process requiring the coordinated action of multiple components, including NEDD8, E1, E2, a multifunctional RING E3, and the cullin substrate. Subsequent to neddylation modification, the Cullin-RING complex, in conjunction with ubiquitin E1, E2, and a plethora of substrate recognition subunits and adaptor proteins, promotes the ubiquitylation of substrate proteins. This assemblage constitutes a diverse, intricate, and finely regulated machinery, involving numerous protein-protein interactions and transient or continual conformational changes. The mechanisms by which neddylation achieves timely and accurate regulation remain largely elusive. Structural biology studies offer the most direct and compelling evidence to unravel these mysteries. A comprehensive elucidation of the aforementioned structures would facilitate structure-based drug design and virtual screening for targeting neddylation enzymes, thereby accelerating the discovery process of small molecule inhibitors.^90^ Table 3 summarizes the published crystal structures of the major components of cullin neddylation. Below we focused on several pivotal structural studies on cullin neddylation.

**Table 3:** Released structures of human neddylation-related proteins

PDB ID *EMDB ID*	Release Year	Method	Resolution (Å)	Components^a^
Neddylation enzymes and cullins alone				
1NDD	1999	X-RAY	1.6	NEDD8
2KO3	2009	NMR		NEDD8
2LQ7	2012	NMR		UBA3
2EDI	2007	NMR		UBE2F
2LGV	2012	NMR		RBX1
2ECL	2007	NMR		RBX2
2MYL, 2MYM	2015	NMR		CUL3
2DO7	2007	NMR		CUL4B
4A64	2012	X-RAY	2.57	CUL4B
8EI1	2023	X-RAY	2.89	CUL4B
8EI2	2023	X-RAY	2.8	CUL5
2JNG	2007	NMR		CUL7
6B5Q, 6BG3, 6BG5	2018	X-RAY	2.16	DCN1
5UFI, 5V83, 5V88, 5V89	2017	X-RAY	2.58	DCN1
NEDD8 in complex				
8CAF	2023	X-RAY	2.66	NEDD8-CUL1-N8C_Fab3b
2N7K	2016	NMR		NEDD8-KHNYN
4FBJ	2012	X-RAY	1.6	Cif-NEDD8
2BKR	2005	X-RAY	1.9	NEDD8-SENP8
1XT9	2004	X-RAY	2.2	NEDD8-SENP8
Neddylation E1s				
3GZN	2010	X-RAY	3	NAE1-UBA3-NEDD8
3DBH, 3DBL, 3DBR	2008	X-RAY	2.85	NAE1-UBA3-NEDD8
1YOV	2005	X-RAY	2.6	NAE1-UBA3
1R4M, 1R4N	2003	X-RAY	3	APPBP1-UBA3-NEDD8
Neddylation E1-E2 complex				
3FN1	2009	X-RAY	2.5	UBA3-UBE2F
2NVU	2007	X-RAY	2.8	APPBP1-UBA3 ~ NEDD8-NEDD8-UBE2M
1Y8X	2005	X-RAY	2.4	UBA3-UBE2M
1TT5	2004	X-RAY	2.6	NAE1-UBA3-UBE2M
Neddylation E2-E3 complex				
8B3I	2023	EM	3.5	NEDD8-CUL4A-RBX1-DDB1-ERCC8-UBE2D2-UB
6TTU	2020	EM	3.7	NEDD8-CUL1-RBX1-SKP1-BTRCP-IKBA-UB ~ UBE2D2
4P5O	2014	X-RAY	3.1071	RBX1-UBC12 ~ NEDD8-CUL1-DCN1
4GBA, 4GAO	2012	X-RAY	2.4	DCUN1D2/3-UBE2F
3TDU, 3TDZ	2011	X-RAY	1.5	DCN1-CUL1-UBE2M
Neddylation E3 complex				
8KHP	2023	EM	3.67	CUL3-KLHL22-RBX1
8QU8	2023	EM	3.5	KRAS-VHL-ELOB-ELOC-CUL2-RBX1
8JAQ, 8JAV, 8JAS	2023	EM	3.26	CUL2-RBX1-ELOB-ELOC-APPBP2
8GQ6, 8H33, 8H34, 8H35, 8H3R, 8H36, 8H37	2023	EM	4.6	KBTBD2-CUL3-RBX1
7Z8B	2022	EM	2.8	CUL7-RBX1-SKP1-FBXW8
7OKQ	2021	EM	8.4	DDB1-DCAF1-CUL4A-RBX1
7ONI	2021	EM	3.4	NEDD8-CUL5-RBX2-ARIH2
7B5N, 7B5S	2021	EM	3.6	NEDD8-CUL1-RBX1-UBE2L3 ~ UB ~ ARIH1
7B5M, 7B5L, 7B5R	2021	EM	3.8	NEDD8-CUL1-RBX1-SKP1-SKP2-CKSHS1-CCNA2-CDK2-CDKN1B
5N4W	2017	X-RAY	3.9	CUL2-RBX1-ELOB-ELOC-VHL
5V89	2017	X-RAY	1.55	DCUN1D4-CUL1
4F52	2012	X-RAY	3	GLMN-RBX1-CUL1
4A0K	2011	X-RAY	5.93	CUL4A-RBX1-DDB1
3RTR	2011	X-RAY	3.21	CUL1-RBX1
3DQV, 3DPL	2008	X-RAY	3	NEDD8-CUL5-RBX1
2HYE	2006	X-RAY	3.1	DDB1-CUL4A-RBX1-SV5V
1LDJ, 1LDK	2002	X-RAY	3	CUL1-RBX1
CAND1 and CSN complex				
8H3Q	2023	EM	3.76	CAND1-CUL3-RBX1
8H38, 8H3A, 8H3F	2023	EM	4.25	KBTBD2-NEDD8-CUL3-RBX1-CSN
8OR2,8OR3	2023	EM	2.9	CAND1-CUL1-RBX1-SKP1-SKP2-DCN1
8OR0, 8OR4	2023	EM	3.1	CAND1-CUL1-RBX1-SKP1-SKP2-CKS1-CDK2
7Z8R, 7Z8T, 7Z8V, 7ZBW, 7ZBZ, ;8CDJ, 8CDK	2023	EM	2.7	CAND1-CUL1-RBX1-SKP1-SKP2
6R7F, 6R7I, 6R7N, 6R7H, 6R6H	2019	EM	8.2	NEDD8-CUL2-RBX1-ELOB-ELOC-VHL-CSN
*EMD-3313,3314,3315,3316*	2016	EM	6.7-8.8	CSN-NEDD8-CUL4A-RBX1
*EMD-3401*	2016	EM	7.2	CSN-NEDD8-CUL1-RBX1-SKP1-SKP2-CKS1
*EMD-2173, 2174,2175*	2012	EM	25	CSN-NEDD8-CUL1-RBX1-SKP1-SKP2-CKS1
4A0C	2011	X-RAY	3.8	CAND1-CUL4B-RBX1
1U6G	2004	X-RAY	3.1	CAND1- CUL1- RBX1

Data source: RCSB Protein Data Bank (https://www.rcsb.org/) and EMDataResource (https://www.emdataresource.org/). If a same structure was released on both RCSB Protein Data Bank and EMDataResource, only PDB ID was provided for space saving. Data were last updated on Jan 7th, 2024

*X-RAY* X-ray diffraction, *NMR* solution NMR, *EM* electron microscopy

^a^The components of each structure are listed with their gene names

**Fig. 3:**
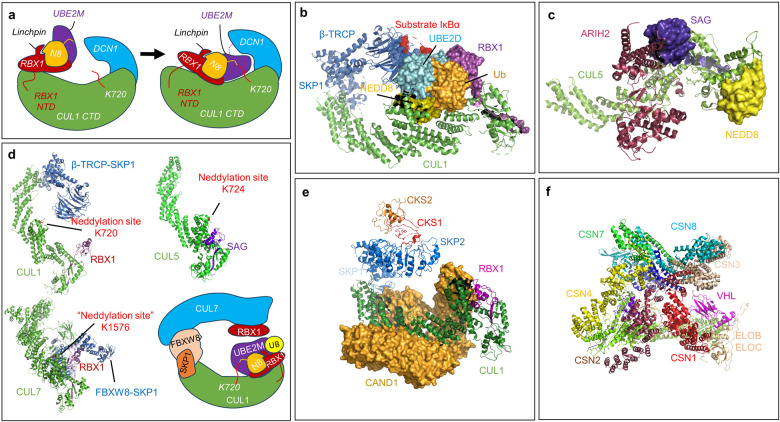
The crystal structures of neddylation enzymes in complex with cullin substrates. **a** The model derived from the co-crystal structure of RBX1-UBE2M ~ NEDD8-CUL1^CTD^-DCN1^P^ revealed how neddylation induces a conformational change to bring the E2 close to the substrate (modified based on the reported structure^96^). **b** The crystal structure of a neddylated CRL1^β-TRCP^-UBE2D~Ub-substrate intermediate revealed how NEDD8 acts as a nexus between cullin elements and the RING-activated ubiquitin-linked E2 to promote substrate ubiquitylation (PDB ID: 6TTU). **c** Co-crystal structure of CUL5-ARIH2 E3-E3 ubiquitin ligase unveiled the unique role of NEDD8 in promoting interactions of CUL5-RBX2 with a RING-between-RING (RBR) E3, ARIH2 (PDB ID: 7ONI). **d** An alignment of the reported structures of CRL1^β-TRCP^, SAG-CUL5, and CRL7^FBXW8^ (PDB ID: 6TTU, 6V9I and 7Z8B) shows the different CRL structures with the reported neddylation site. A model of CRL7 in complex with neddylated CRL1 illustrates a unique CRL-CRL interaction that enhances substrate ubiquitylation. **e** The Electron microscopy structure of CAND1-CUL1-RBX1-SKP1-SKP2-CKS1-CDK2 complex (PDB ID: 8OR0). **f** The Electron microscopy structure of NEDD8-CUL2-RBX1-ELOB-ELOC-VHL-CSN (PDB ID: 6R7F). All protein structures were illustrated by PyMol software (The PyMol Molecular Graphics System, Version 2.6.0a0 Open-Source, Schrödinger, LLC)

While early structural biological studies have revealed basic mechanisms of substrate ubiquitylation mediated by CRL, a gap of approximately 50 amino acids was observed between the CRL-bound substrate and the active-site cysteine of a E2.^91–93^ Based on extensive biochemical assays and enzymatic reactions using purified proteins, NEDD8 was proposed to play a crucial role in closing this 50 Å gap.^94^ In 2008, a crystal structure of the neddylated CUL5^CTD^-RBX1 was resolved by the Schulman group, revealing that neddylation induces a significant change in conformation, pushing RBX1 and its bound activated E2 enzyme into a close conformation, thus bridging the 50 Å gap to bring the E2 ~ Ub near the substrate.^95^ Later in 2014, the same group unveiled the first co-crystal structure of a RING E3-E2 ~ NEDD8-CUL1^CTD^-DCN1P at 3.1 Å, providing intricate insights into mechanisms that govern NEDD8 ligation and specificity.^96^ This crystal structure of CUL1-RBX1 in complex with NEDD8-conjugated UBE2M revealed how cullin neddylation activates CRLs. Specifically, the UBE2M ~ NEDD8 is anchored to the RBX1 RING domain and forms a compact structure that is stabilized by a “linchpin” arginine residue unique to RBX1 (Arg46). Notably, the C terminus of NEDD8 is covalently linked to a Ser residue, replacing the catalytic Cys111 in the modified UBE2M. The oxyester-bonded UBE2M ~ NEDD8 intermediate binds to a specific linker sequence that bridges the initial beta-strand of the RBX1 protein with its RING domain. This precise interaction aligns the NEDD8 transfer module in an optimal orientation, ensuring that the catalytic site of the NEDD8 E2 enzyme is situated adjacent to the neddylation site Lys720 on CUL1. Additionally, it is positioned near the WHB subdomain, which is detailed in Fig. 3a. This model further highlights the importance of the multifunctional RING E3s with regards to the specificity in neddylation modifications.

Subsequent studies provided a more comprehensive view of the concerted mechanism of ubiquitylation and neddylation, revealing numerous NEDD8-dependent inter-protein interactions. In 2020, a cryo-electron microscopy structure of a neddylated CRL1β-TRCP-UBE2D~Ub-substrate intermediate showed how NEDD8 acts as a nexus between cullin component and ubiquitin-loaded E2 activated by the RING to promote substrate ubiquitylation.^97^ Based on structural remodeling, the study showed that numerous interactions among NEDD8, the cullin and RING-bound E2~Ub intermediates for rapid substrate ubiquitylation (Fig. 3b), and the specificity of neddylation RING E3s in complex with specific cullin partners.^97^

Recent structural and biochemical studies have discerned significant differences between the NEDD8/CUL1 (or CUL1-4, 7, and 9) and NEDD8/CUL5 interactions,^95,97–99^ although the exact mechanism governing the specific arrangement of the two RING E3s, RBX1 and RBX2, in neddylation remains elusive. Nevertheless, a few recent structural biology studies provide some mechanistic insights. For example, co-crystal structure of CUL5-ARIH2 E3-E3 ubiquitin ligase unveiled the unique role of NEDD8 in promoting interactions of CUL5-RBX2 with a RING-between-RING (RBR) E3 ARIH2.^100^ In that reported structure of NEDD8/CUL5^CTD^/RBX2/ARIH2 complex (Fig. 3c), the surface of NEDD8 interacts differently with CUL5, as compared to CUL1. The Leu710 and Glu717 on CUL5 bind to Ile44 patch on NEDD8, while the corresponding CUL1 residues Asp and Ala, which are conserved across CUL1-4, are incompatible with the Ile44 patch on NEDD8. This results in a distinct structural arrangement, highlighting the cullin-specific regulation by NEDD8. Furthermore, the study also uncovered an allosteric mechanism as to how the unique CUL5-ARIH2 E3-E3 interaction is induced by NEDD8,^100^ potentially paving the road for future discovery of allosteric drugs (Fig. 3c).

Earlier structural biology studies, including mutation experiments, have revealed that a specific lysine residue on the WHB domain of cullins (K720 on CUL1^96^ and K724 on CUL5^100^) serves as the neddylation site (Fig. 3d, top two panels). In a recent study, the cryo-EM and biochemical analyses were used to resolve the structure of CRL7-FBXW8 complex, which shows a distinctive mechanism of forming a specific CUL-RBX partnership.^101^ Specifically, the CRL7-FBXW8 complex adopts a T-shaped structure, which is characterized by CUL7–RBX1 forming a long bar that intersects the CUL7-FBXW8–SKP1 base at a significant angle. This unique arrangement is indicative of the distinct architecture of complex.^101^ However, despite the presence of a corresponding lysine residue on CUL7 (K1576), structural differences result in this site being fixed and buried, rendering CUL7 incapable of undergoing neddylation (Fig. 3d, bottom left panel). Moreover, contrary to CUL1-RBX1, CUL7–RBX1 in the CRL7-FBXW8 complex does not exhibit auto-ubiquitylation activity under conditions where CUL1-RBX1 complexes effectively auto-ubiquitinate. These observations align with the structural findings, revealing a restricted orientation of RBX1 RING domain within CRL7-FBXW8. The N-terminal region of RBX1 forms a β-strand within the α/β region of CUL7, creating an intermolecular cullin/RBX domain crucial for the stability of the complex.^101^ And this unique structure prevents the binding of the RBX1 RING domain to an active E2 ~ NEDD8 or E2~ubiquitin intermediate, leading to the lack of auto-neddylation and ubiquitylation activities in the purified CRL7-FBXW8 complex. More intriguingly, CRL7-FBXW8 could form a complex with neddylated CUL1-RBX1, presenting a special CRL-CRL partnership. This duo-CRL complex facilitates full E3 ligase activity and therefore promotes substrate ubiquitylation, even if the CRL7-FBXW8 is not neddylated^101^ (Fig. 3d bottom right panel). However, any other involving mechanism or whether RBX1 plays an additional role within this complex remain elusive.

While the co-crystal structure of CUL1/RBX1 and CUL5/RBX2 has both been resolved, the scenario is quite different when comes to the E2-RING partners. Surprisingly, although the co-crystal structure of the RBX1-UBC12 complex was resolved 10 years ago,^96^ no co-crystal structure of the RBX2-UBE2F complex was reported so far. Additional effort should be made to resolve the structure of this specific neddylation enzyme complex to elucidate the underlying mechanism related to the specificity of CUL5 neddylation, which should pave the road for structure-based design and virtual screening to discover small molecule inhibitors of the RBX2-UBE2F complex. Furthermore, the co-crystal structures of various neddylation E3 with their non-cullin substrates are also lacking, likely due to the lack of solid evidence that the pair of given E3-substrate is physiologically relevant in vivo.

CAND1 and CSN are crucial components in neddylation, either as a negative regulator by selectively binding to unneddylated CUL1-RBX1, thus blocking neddylation, or reversing the neddylation reaction via removal of NEDD8 from neddylated substrates, respectively.^102^

The early X-ray crystallographic as well as kinetic studies on the effect of CAND1 binding to CUL1/RBX1 or CUL4/RBX1 suggested a model where CAND1 binds to unneddylated cullins, facilitating the removal of substrate receptor (SR) modules like SKP1-SKP2.^87,103^ However, the detailed mechanism of how CAND1 drives the reaction of SR exchange remained elusive. Most recently, the cryo-EM structure of a substrate-bound CAND1-SCF complex, comprising CAND1-CUL1-RBX1/SKP1/SKP2/CKS1-CDK2, was resolved at 3.1 Å resolution,^104^ which demonstrates the dynamic interaction of CAND1 with CUL1/RBX1 and SRs (Fig. 3e). Consistently, the Schulman group published a series of cryo-EM structures of the CAND1-SCF complex, illustrating the assembly and disassembly processes of CAND1 and SRs, and these dynamic conformational changes regulate the activity of SCF/CRL1 E3 complexes.^105^

The CSN deneddylase consists of eight subunits (CSN1-8), with CSN5 serving as the catalytic subunit.^83^ A series of EM structures of CSN-CRL complexes have been released, including CSN in complex with CRL1, CRL2, CRL3 and CRL4A,^85,106,107^ and the cryo-EM structures of the NEDD8-CRL2-CSN^108^ (Fig. 3f). These structures data suggest a relatively conserved mechanism for CSN deneddylation, involving at least three steps: 1) CSN2 and CSN4 initiate the process through conformational clamping of the CRL substrate, 2) the CSN5/CSN6 heterodimer is then released, and 3) CSN5 activates the deneddylation machinery.^83,85,106–108^

Despite substantial progress has been made in understanding the functions of CAND1 and CSN in neddylation, the intricate details of the mechanism remain largely unknown. The structural versatility of CAND1, coupled with the inherent flexibility of CRL structures, suggests a wide range of possible conformations, possibly tailored to specific cullins. Furthermore, the dynamic and complex nature of CSN binding and its deneddylation process remains ambiguous. The future studies are geared toward in-depth structural and kinetic studies to elucidate the underlying mechanisms by which CAND1-mediated exchanges of CRL substrate receptors and the CSN-driven CRL deneddylation.

## Neddylation in health: regulation of biochemical reactions and biological processes

By targeting numerous cullin and non-cullin proteins, neddylation pathway is able to directly or indirectly regulate multiple signaling transduction pathways involved in a variety of biological processes, including cell cycle and cell survival, cell metabolism, DNA damage responses and repair, cellular immune-responses, among others.^17,109^ Thus, precise regulation of neddylation pathway is crucial for the maintenance of the homeostasis of a cell to ensure a healthy operation.

### The role in signaling transduction and epigenetic regulation

#### The NF-κB signal

Nuclear factor-kappa B (NF-κB) is a transcription factor serving as a hub for transactivation of a variety of proteins which are essential for cell survival and immune responses.^110^ Two pathways known to activate NF-κB are the canonical and the non-canonical pathways. In canonical pathway, NF-κB is activated upon induced ubiquitylation and degradation of the inhibitor of nuclear factor kappa B (IκB).^110^ Upon several stimuli, such as TNFα or LPS, IκB is phosphorylated by a heterotrimeric kinase composing of IKKα, IKKβ, and IKKγ, followed by targeted ubiquitylation and degradation by SCF^βTrCP^ (CRL1^βTrCP^) E3 ligase. The removal of IκB triggers p65 nucleus translocation where NF-κB (complex of p50/p65) transactivates the expression of many genes.^111^ Neddylation of CUL1 activates SCF^βTrCP^ which is required for optimal ubiquitylation and degradation of IκB.^112^ Another example of neddylation activation of the NFκB signal was observed through CRL5. Upon LPS exposure, NEDD8 is recruited and neddylated CUL5 to activate CRL5, which promotes polyubiquitylation of tumor necrosis factor receptor-associated factor 6 (TRAF6) via the K63 linkage to activate NF-κB and induced expression of proinflammatory cytokines in bone marrow-derived macrophages (BMDMs).^113^ Interestingly, NF-κB is also subjected to neddylation-mediated negative regulation. It was shown that the IKKγ is neddylated by RING E3 ligase TRIM40, leading to inhibition of kinase activity of the IKK complex, thus preventing IκB from degradation and NF-κB from activation, which appears to be an important mechanism to prevent inflammation-associated carcinogenesis in gastrointestinal epithelium.^45^

The NF-κB-inducing kinase (NIK) is a central signaling component of the noncanonical NF-κB pathway. In response to some stimuli, such as the ligands of TNFR superfamily members, NIK acts together with the IKKα to trigger phosphorylation-dependent processing of p100 for its proteolytic cleavage, generating the mature p52 form as the non-canonical NF-κB2.^114^ Importantly, NIK was recently reported as a substrate, subjecting to neddylation, which triggers its degradation.^115^ Hepatic-specific *Nae1* deletion that inhibits its neddylation causes NIK accumulation and aberrant activation, resulting in activation of non-canonical NF-κB pathway, contributing to inflammation and hepatocyte damage.^115^

### The HIF‐1α signal

Hypoxia-inducible factor 1 (HIF-1), a heterodimer consisting of an oxygen inducible factor (HIF-1α) and the constitutive HIF-1β (AhR nuclear translocator, ARNT), is a paramount transcription factor that upregulates a wide range of hypoxia-inducible genes, and acts as a signaling hub.^116^ Under normoxic conditions, HIF‐1α is hydroxylated by prolyl hydroxylase domain (PHD) proteins, then is recognized by the von Hippel-Lindau (VHL) protein for ubiquitylation and subsequent proteasomal degradation.^117^ VHL is a substrate-receptor subunit of CRL2, composing of CUL2, elongins B and C, and RBX1.^118^ Importantly, neddylation of CUL2 activates CRL2^VHL^ to ubiquitylate HIFα.^119^ Interestingly, the recognition of HIFα by VHL is essential for triggering RBX1-mediated CUL2 neddylation, since HIF-1α mutation on the VHL binding residues reduces CUL2 neddylation.^119,120^ MLN4924, a small molecule inhibitor of neddylation E1,^18^ inhibits CUL2 neddylation, leading to stabilization of HIF-1α.^121,122^ Moreover, VHL itself can be neddylated, possibly mediated by MDM2, which precludes CRL2 complex formation to recruit HIF-1α.^123,124^ Interestingly, our previous study revealed that UBE2M is subjected to HIF-1 transactivation upon exposure to hypoxia, whereas MLN4924 increases UBE2M expression via inhibiting HIF‐1α degradation.^55^ Thus, HIF‐1α also regulates the expression of neddylation enzyme, not just as a substrate of CRL2 upon neddylation activation.

### Receptor tyrosine kinases and MAPK signals

Receptor tyrosine kinases are cell surface receptors which are activated upon binding to their ligands such as growth factors, cytokines, and hormones to trigger phosphorylation and activation of the downstream proteins important for cell proliferation, differentiation and metabolism.^125^ Transforming growth factor β type II receptor (TGFβRII) and epidermal growth factor receptor (EGFR) are two examples of RTK signaling subjected to NEDD8 regulation. TGF-βRII has potent anti-proliferative effect in multiple types of cells, and deregulation of TGF-β signaling is highly implicated in tumorigenesis.^126^ The c-Cbl E3 was identified as a NEDD8 E3 ligase of TGFβRII that promotes conjugation of NEDD8 to TGFβRII at Lys556 and Lys567.^47^ The neddylated TGFβRII keeps itself from endocytosis to caveolin-positive compartments, leading to a prolonged stability,^47^ whereas deneddylation of TGFβRII by DEN1/NEDP1/SENP8, one of NEDD8 isopepitidases, results in TGFβRII ubiquitylation and degradation through lipid caveolin-mediated endocytosis.^47^ Finally, aberrant TβRII neddylation appears to be associated with leukemia development, since the c-Cbl neddylation-dead mutation with potential low levels of TGF-βRII neddylation is a common event in leukemia.^47^

EGFR receptor tyrosine kinase, upon binding to extracellular growth factors or growth hormones, is activated through dimerization to trigger its intrinsic intracellular protein-tyrosine kinase activity, followed by activation of numerous down signaling cascades in cells.^127^ The c-Cbl is a well-known ubiquitin E3 ligase to target EGFR for ubiquitylation, which serves as a sorting signal for endocytosis and lysosomal degradation.^128^ Interestingly, the c-Cbl also acts as an NEDD8 E3 to promote EGFR neddylation, contributing to subsequent EGFR ubiquitylation and endocytic internalization.^129^ HER2 belongs to the family of EGFR, which is phosphorylated upon binding to growth factors to activate downstream kinases for intracellular signal transduction.^130^ HER2 was recently reported as a neddylation substrate, and neddylation modification reduces UPS-mediated degradation, leading to its stabilization.^131^

The neddylation pathway was also found to be required for the activation of the RAS/ERK signal.^132^ Specifically, the SHC, an adaptor protein bridging the antigen receptor and the RAS/ERK signal, was reported to be a neddylation substrate, and the SHC neddylation at lysine 3 mediated by UBE2M facilitates the formation of a ZAP-SHC-GRB2 signaling complex, which modulate the downstream ERK activation and aggravation of inflammation.^132^ Furthermore, a recent study reported that KDM2A, a lysine demethylase, promotes the proteasomal degradation of transcription factors TCF/LEF in a manner independent of its demethylase domain. This effect is blocked by neddylation inhibitor MLN4924, suggesting that neddylation also regulates the canonical Wnt signaling.^133^ Taken together, neddylation regulates a number of important signal transduction pathways, highlighting its importance in cell growth and proliferation.

### Epigenetic regulation

Epigenetic regulation is referred to a variety of covalent modifications of nucleic acids and histone proteins, which can heritably and reversibly modify gene expression and chromatin structure without affecting DNA sequence.^134^ Specifically, epigenetic regulation can occur via DNA methylation, histone modifications, chromatin remodeling, and noncoding RNA modulation,^134^ which play essential role in various biological processes, and dysregulation of which is associated with few human diseases.^135–137^ Notably, NEDD8-conjugated protein appears to be critical for DNA methyltransferases 3b (DNMT3b)-dependent DNA methylation, since DNMT3b interacts with the neddylated form of CUL4A preferentially, and inactivation of NAE1 inhibits DNMT3b-dependent DNA methylation.^138^ The neddylation-controlled ubiquitin E3 ligase CUL4^DDB1^ could bind to WDR5 and RBBP5, and regulate histone H3K4 methylation.^139^ Moreover, CUL4 is also associated with Clr4, a histone methyltransferase in *S. pombe* to promote lysine 9 methylation on histone H3, a critical process to heterochromatin formation.^140^ Histone deacetylases (HDACs) are enzymes that remove acetyl groups from histones, making a more tight wrap of histones around the DNA, thus inhibiting gene transcription.^141^ Neddylation pathway controls the activity of HDACs, since NEDD8 facilitated the ubiquitylation and clearance of HDAC1 and HDAC2,^142,143^ and inhibition of NEDDylation by MLN4924 increases protein levels of HDAC4/5,^144^ and significantly increases HDAC6 activity.^145^

### The role in stress responses

#### DNA damage response

DNA double-strand breaks are common lesions that cause genomic instability, cell death and tumorigenesis when left unrepaired.^146^ In response to DNA damage, p53 is phosphorylated within the MDM2-binding motif that disrupts the p53-MDM2 binding and MDM2-mediated degradation. Increased p53 then induces cell cycle arrest to trigger DNA repair process or causes apoptosis, if the damage is too severe.^147^ Interestingly, p53 was subjected to neddylation modification by MDM2 as a part of cellular response to UV radiation in breast cancer cells.^43^ Furthermore, MDM2 can be self-neddylated in a way similar to its autoubiquitylation,^43^ and the neddylated MDM2 maintains its protein stability to keep p53 level in check.^148^ On the other hand, the NEDD8-specific isopeptidase DEN1 is significantly induced during DNA damage response, leading to MDM2 deneddylation and destabilization, and p53 activation.^148^

The transcription factor E2F-1 is also induced in analogous to p53 in response to DNA damage.^149^ While the E2F-1 activity is usually suppressed by neddylation modification, and the neddylated E2F-1 levels appear to decrease by ~80% upon etoposide or doxorubicin treatment, suggesting that E2F-1 neddylation is strictly regulated in response to DNA damage.^150^ DNA-dependent serine/threonine protein kinase catalytic subunit (DNA-PKcs) is a heterotrimeric protein kinase that initiates the signaling cascades in the non-homologous end-joining (NHEJ) repair upon DNA damage.^151^ DNA-PKcs was reported to be neddylated by the NAE-UBE2M-HUWE1 cascade, which is required for DNA-PKcs activation, since blockage of DNA-PKcs neddylation by MLN4924 or depletion of HUWE1 inhibits the autophosphorylation of DNA-PKcs and lessens the efficiency of NHEJ.^152^ Consistently, we previously showed that CRL1/SCF^FBXW7^ E3 promotes polyubiquitylation of XRCC4 at K296 via the K63 linkage to facilitate NHEJ repair process. Inactivation of SCF^FBXW7^ by MLN4924 blocks this process, abrogates NHEJ and renders cancer cells much more sensitive to radiation.^153^

In another interesting study, NEDD8 was found to be accumulated and co-localized at DNA damage sites upon DNA damage stimuli, followed by conjugation of NEDD8 chains to the N-terminal lysine residues of histone H4, catalyzed by the UBE2M-RNF111 cascade. Such polyneddylation chain induced by DNA damage is recognized by the UIM (motif interacting with ubiquitin) domain of another RING E3 ligase, RNF168 to recruit BRCA1 and 53BP1, two homologous recombination repair factors, thus amplifying the DNA damage response cascade.^44^ Besides, RNF168 mediates both ubiquitylation and neddylation of H2A. Upon DNA damage, H2A neddylation is inhibited, whereas its ubiquitylation is induced, which facilitates DNA damage repair.^154^ Finally, RNF168 itself is neddylated, which activates its E3 ligase activity.^154^

Interestingly, there is a balance between mono- and poly-neddylation, which is sensed by Heat Shock Protein 70 (HSP70). Upon DNA damage, NEDP1 is induced to convert unanchored poly-NEDD8 chains to mono-NEDD8 in HSP70, which activates ATPase activity of HSP70, and promotes the formation of APAF1 oligomerization to induce apoptosis.^61^ Furthermore, non-cullin conjugates poly-NEDD8 chains were detectable in cells, which are stabilized upon oxidative stress or when NEDP1 is depleted. Moreover, an unanchored, trimeric NEDD8 interacts with poly(ADP-ribose) polymerase 1 (PARP-1), and to attenuate its activation, thus protecting cell death induced by oxidative stress.^62^ Taken together, neddylation pathway appears to play a significant role in modulating DNA damage responses.

#### Nucleolar stress

Ribosome biogenesis is an active nucleolar process that consumes large amount of the cellular energy, thus is regulated tightly.^155^ Impaired ribosome assembly upon harmful stimuli could cause cell cycle arrest by activated p53, given several ribosomal proteins are capable of interacting with MDM2 to disrupt the MDM2-p53 binding.^156^ Interestingly, neddylation-deneddylation of ribosomal protein L11 (RPL11) regulates its subcellular localization and stability. Specifically, MDM2 acts as an NEDD8 E3 to directly neddylate RPL11, leading to its stabilization and nucleolus localization, whereas deneddylation by DEN1 results in its relocalization from nucleolus to the nucleoplasm where RPL11 is degraded by an unknown E3 ligase.^157,158^ Upon nucleolar stress, the neddylated RPL11 is rapidly located to the promoter sites of p53 target genes through interacting with MDM2, and consequently enhances p53 transactivation activity.^159^ Likewise, upon nucleolar stress the ribosomal protein S14 (RPS14) is neddylated and stabilized to induce p53 by inhibiting MDM2-mediated p53 degradation.^160^ In addition, MDM2 and DEN1 also mediate neddylation and de-neddylation of the ribosomal protein S27 and RPS27L, respectively, which regulate their stability and survival of cancer cells.^161^ More interestingly, MDM2 acts as a dual E3 acting either as ubiquitin E3 to degrade p53 or as neddylation E3 to inhibit transcriptional activity of p53.^43^ Similarly, the ubiquitin ligase SCF^FBXO11^ also promotes p53 neddylation, and suppresses its transcriptional activity.^162^ Taken together, ribosomal stress triggers p53 activation via modulating the MDM-RPs axis and neddylation plays a significant role in the process. Given p53 neddylation suppresses its transcriptional activity, treatment of cancer cells harboring a wt p53 with neddylation inhibitor would, in principle, reactivate p53 to induce growth arrest or apoptosis.

### The role in development

NEDD8 was first isolated from the mouse brain as a possible regulator for the differentiation and development of the central nervous system.^25^ Interestingly, while Nedd8 knockout stain is available (https://www.informatics.jax.org/marker/MGI:97301), no study was reported in PubMed to define the phenotypes of Nedd8 total knockout, although it is expected to be embryonically lethal. In fact, our unpublished data showed that it is indeed the case; the Nedd8-null embryos die before E10.5. Likewise, the mice with total knockout of *Uba3* die in utero at the stage of peri-implantation. Furthermore, Uba3-null trophoblastic cells fail to enter the S phase of the endoreduplication cycle with aberrant expression of cyclin E and p57.^163^

Several studies on tissue-specific knockout of neddylation E1 were conducted. Specifically, the conditional KO of Nae1 in the cortical progenitors causes premature death at the postnatal day (P) 28 with disorganized cerebral cortex, as evidence by thinner cortex and enlarged lateral ventricle, similar to the phenotypes observed in mutant mice with gain-of-function of β-catenin, as confirmed by a rescue experiment via suppressing β-catenin expression, indicating the Wnt/β-catenin signaling is subject to neddylation regulation in vivo.^164^ For postnatal brain development, neddylation pathway is also critical, given that mice with cKO of *Nae1* in forebrain excitatory neurons in adulthood led to synaptic instability, impaired neurotransmission and cognitive deficits.^165^ On the other hand, a study using primary rat cultured neurons showed that the expression of deneddylating enzyme DEN1/SENP8/NEDP1 reaches the peak around the first postnatal week and gradually diminishes in mature brains and neurons. In fact, DEN1 negatively regulates neurite outgrowth and plays an important role in neuronal development via targeting a number of signaling pathways, including actin dynamics, Wnt/β-catenin, and autophagic processes.^166^

Neddylation pathway also regulates the heart development. Both neddylation enzymes and neddylated proteins are expressed highly in embryonic hearts, but remarkably downregulated after birth.^167^ Targeted deletion of *Nae1* in mouse cardiomyocytes results in myocardial hypoplasia and ventricular non-compaction, and eventually causes heart failure and perinatal lethality, demonstrating its critical role during the development of cardiac chamber in embryonic hearts.^167^ For postnatal cardiac development, neddylation is required for proper control of metabolic transition in cardiomyocytes by regulating the conversion between glycolysis and oxidative metabolisms. Postnatal deletion of *Nae1* in mice hearts causes disruption of transverse tubule formation and repression of fetal-to-adult isoform switching, leading to immature cardiomyocytes, and eventually cardiomyopathy and heart failure.^168^

The PubMed search did not yield any publications on the total knockout studies of neddylation E2s Ube2f or Ube2m in mice, although it is again expected to be embryonically lethal. In fact, we have attempted to generate a KI mouse model with enzymatic dead mutant (C116A) of Ube2f, but unable to obtain homozygous KI alleles. Further careful examining the KI construct revealed an error in design that fails to generate desired KI, rather a total KO mouse, suggesting that total KO of Ube2f in mice is embryonic lethal as expected (unpublished data).

For two neddylation E3s, Rbx1 and Sag/Rbx2, required for cullin neddylaiton, our earlier studies showed total knockout either caused the embryonic lethality without mutual compensations, indicating their non-redundant role.^169,170^ Specifically, *Rbx1* total knockout caused embryonic death at the E6.5 with remarkable proliferation defects due to p27 accumulation. Simultaneous *p27* total KO extends the embryo life to E9.5, indicating accumulation of other critical substrates upon *Rbx1* knockout play the essential role during the late-stage development.^169^ The Sag/Rbx2 total knockout also causes embryonic death at E11.5–12.5 with severe defectives in vascular and nervous system. Mechanistically, some developing organs in the embryos at the stage E8.5-9.5 undergo hypoxia stress,^171^ which induces Sag^172^ to recruit Fbxw7 for ubiquitylation and degradation of neurofibromatosis type I (NF1),^170^ a natural occurring inhibitor of Ras, leading to the activation of MAPK signals to promote proliferation, vasculogenesis, angiogenesis and inhibit apoptosis. Sag knockout causes Nf-1 accumulation to block the Mapk signals, leading to defective proliferation, vasculogenesis, and massive apoptosis. We found that simultaneous *Nf1* knockout improves the defects in vasculogenesis, but without extending the lifespan of the embryoes,^170^ suggesting additional substrates play essential role for the survival. Finally, Tie-Cre driven endothelial *Sag* deletion also caused embryonic lethality at E15.5 again with poor vasculogenesis.^173^

Taken together, the neddylation pathway, including its component Nedd8, and its catalyzing enzymes (neddylation E1/2/3) are all essential for development of mouse embryos as well as mouse organs, emphasizing the functional importance of this modification.

### The role of immune system

Accumulated data have revealed that inhibition of neddylation pathway via genetic or pharmacological approaches effectively affects cellular immune responses and the functionality of immune cells.^22^ Indeed, the expression of several components of neddylation complex are markedly altered upon exposure to immune stimuli. For example, the levels of both mRNA and protein of RBX2 are increased ~10 folds in macrophage J774 cells after stimulation with lipopolysaccharides (LPS) or polyinosinic-polycytidylic acid (polyI:C).^174^ The neddylation of CUL1 is apparently increased in LPS-stimulated human microvascular endothelial cells.^175^ Moreover, Nedd8 knockout in zebrafish via CRISPR/Cas9 significantly inhibits the expression of key antiviral genes after polyI:C stimulation or SVCV (Spring Viremia of Carp Virus) infection, leading to remarkable increases in SVCV replication, indicating a critical role of neddylation in the antiviral innate immunity.^176^ Likewise, mice with Ube2m KO in myeloid lineage showed severe defects in innate immune response, and were notably sensitive to infection with Herpes Simplex Virus-1 (HSV-1),^177^ and RNA viruses including VSV (vesicular stomatitis virus) and H1N1 virus.^178^ Thus, neddylation pathway is critical for the immune responses, and its abnormal activation or depletion affects the proliferation and function of diverse immune cells, including neutrophils, macrophages, T cells, and Treg cells as described in more details below.^179^

#### Neddylation regulation in neutrophils

Neutrophils is one type of polymorphonuclear leukocytes that are recruited to the inflammatory site to eliminate pathogens, thus acting as the first line of defense in innate immune response.^180^ Neddylation is critically involved in the regulation of neutrophils. Deficiency of Cul5 attenuated alveolar neutrophil recruitment and reduced lung injury in mice receiving intratracheal instillation of LPS.^113^ Mice with *Sag* deletion at the myeloid lineage (via LyzM-Cre) are more susceptible to LPS exposure with high mortality. Mechanistically, in response to LPS, the Sag-deficient CD11b+Gr-1+-neutrophils released more TNFα with significantly decreased expression of myeloperoxidase (Mpo), a neutrophil enzyme, and Elane, a neutrophil expressed elastase.^181^ Neddylation blockage by MLN4924 led to suppression of neutrophil function by inhibiting the NF-κB signal to reduce the production of TNF-α, IL-6, and IL-1β induced by LPS.^182^ Another study reported that the in vivo treatment with MLN4924 increased neutrophil counts in blood, which could be explained by the overcompensation for early-stage anti-inflammatory effects of MLN4924 on endothelium.^183^ Moreover, MLN4924 treatment reduced ischemic brain injury via reducing neutrophil extravasation and maintaining the integrity of blood–brain barrier in mice.^184^

#### Neddylation regulation in macrophages

Neddylation pathway appears to regulate the survival of macrophages, since MLN4924 significantly induces the arrest cells at the G2 phase, and apoptosis in RAW264.7 macrophages.^185^ In addition, neddylation pathway also determines the inflammatory responses of macrophages. Depletion of NEDD8 apparently represses LPS-induced secretion of proinflammatory cytokines (e.g., TNF-α, IL-1β, CXCL1, and IL-6) from macrophages by inhibiting IκB degradation to inactivate NF-κB, thus blocking the transactivation of these cytokine expression.^113,186^ Similarly, RBX2/SAG knockdown induces apoptosis in macrophages, resulting from the accumulation of SAG/E3-regulated pro-apoptotic proteins (e.g., BAX, SARM) when challenged by pathogen-associated molecular patterns (PAMPs), whereas SAG overexpression in BMDMs facilitates the production of proinflammatory cytokines (IL-1β, IL-6, and TNF-α) and concordantly downregulates anti-inflammatory cytokine (IL-10).^174^ Moreover, SAG-deficient macrophages have impaired production of proinflammatory cytokines in response to LPS stimulation.^181^

A recent study revealed that Ube2m is essential to obesity-related inflammation induced by macrophages. Specifically, the mice with macrophage depletion of *Ube2m* have alleviated levels of insulin resistance, obesity, and hepatic steatosis induced by a high-fat diet. Mechanistically, Ube2m depletion blocks the neddylation of TRIM21, an E3 ligase, leading to reduced degradation of VHL. Increased VHL promotes HIF-1α degradation to reduce IL-1β levels.^187^ Paradoxically, while elevated neddylation pathway promotes the infiltration of tumor-associated macrophages and forms a tumor-promoting microenvironment by activating the NF-κB-CCL2 signal in lung cancer cells,^188^ neddylation inhibition also promotes M2 macrophage infiltration and worsens the inflammatory microenvironment by activating the HIF1α-CCL5 signaling in chronic pancreatitis.^189^ Moreover, neddylation enhancement by CSN5 deletion promotes the production of proinflammatory cytokines in macrophages under inflammatory environment by regulating the NF-κB activity, also highlighting the critical role of neddylation pathway in inflammatory responses of macrophages.^183^

#### Neddylation regulation in dendritic cells (DCs)

Neddylation pathway is essential to the survival and activity of DCs, which produce several cytokines and co-stimulatory molecules to strengthen T cell activation.^22,190,191^ During the Mycobacterium tuberculosis infection, CUL1 neddylation is increased in DCs, whereas NEDD8 knockdown promotes phagolysosome fusion in the infected DCs with increased ROS levels, eventually inducing autophagy and apoptosis.^192^ Similarly, inactivation of neddylation pathway also suppresses the survival of DCs by triggering apoptosis or necroptosis through the caspase-dependent manner.^193,194^ Moreover, RBX2 depletion or MLN4924 treatment remarkably inhibits the secretion of proinflammatory cytokines and co-stimulatory molecules, such as TNF-α, IL-6, IL-12p70, thus abrogating the ability of DCs to induce proliferation and activation of T cells.^191,193,194^ Mechanically, neddylation inhibition causes the DEPTOR accumulation by inactivating the CRL-1,^170,193,195^ therefore dampening the mTOR signaling to repress the functions of DCs.^193,196,197^ Furthermore, MLN4924 treatment or SAG knockdown also causes the IκBα accumulation through inactivation of CRL-1, thus inhibiting LPS-induced production of cytokines and blocking the functions of DCs.^191^

#### Neddylation regulation in T cells

T cell activation mediated by the engagement of T-cell antigen receptor (TCR) and co-stimulatory molecules will eventually cause proliferation, cytokine production and differentiation into T helper cells, which is essential for immune response, particularly for antitumor immune response.^198,199^ neddylation blockage by MLN4924 or UBE2M knockdown suppresses proliferation and cytokine production of CD4 + T cells induced by T-cell receptor/CD28 both in vitro and in vivo.^132^ Furthermore, UBA3 depletion compromises the production of antigen-driven cytokines (e.g., IFN-γ, IL-2 and IL-4) in of CD4 + T cells, and impairs the differentiation of follicular helper T (Tfh) cells.^132,200^ Although Sag deficiency has less effect on T-cell development, it apparently attenuates T-cell responses upon in vitro allogeneic stimulation and remarkably reduces graft-versus-host disease.^201^ Mechanically, neddylation pathway appears to directly modulate the adaptor protein Shc to facilitate the formation of a ZAP70-Shc-Grb2 complex, thus inducing the downstream Erk signal,^132,202^ which is essential for T-cell development.^203^ In CD4 + T cells, MLN4924 treatment or UBA3 knockdown profoundly impairs Erk1/2 activation, and dampens differentiation and function.^132^ Moreover, Bcl-2 induction is remarkably inhibited in UBA3-deficient CD4 + T cells upon TCR activation, which causes higher percentage of cell undergoing apoptosis, implying that neddylation pathway regulates the survival of CD4 + T cells through upregulating Bcl-2-mediated apoptosis resistance.^200^ Moreover, neddylation pathway appears to affect the homeostasis of double-negative (CD3 + CD4-CD8-, DN) T cells in a lupus-prone mouse model. Specifically, conditional knockout *Ube2m* in DN-T cells, or treatment with MLN4924 caused accumulation of Bim by blocking its ubiquitylation and degradation to induce apoptosis, leading to decreased DN T cell population and reduced development of murine lupus.^204^ Furthermore, it was reported that during P. yoelii 17XNL infection, neddylation pathway facilitates differentiation of Tfh cells by promoting the neddylation and activation of ubiquitin ligase Itch, which degrades the substrate FoxO1 protein.^200,205^ Thus, UBA3 knockdown greatly inhibits differentiation of Tfh cells by inactivating the ubiquitin ligase Itch.^200,205^ Finally, our early collaborative work showed that Sag-deficient mature naïve T cells is linked to reduced cell proliferation, mitigated cytokine secretion, as well as decreased ability to induce graft-versus-host disease in recipient mice with a mechanism involving the accumulation of SOCS1 and SOCS3 (suppressor of cytokine signaling) proteins.^201^

Regulatory T cells (Treg cells) are a subpopulation of CD4 + T cells which negatively regulate other type of cells in the immune system, thus playing a pivotal role in the maintenance of immunological self-tolerance. It is well-accepted that transcription factor Forkhead box P3 (Foxp3) acts as the master regulator of Treg cells.^206^ To determine the role of neddylation E2s or E3s in functional regulation of Treg cells, we generated the mice with T cell deficiency of either neddylation E2s, *Ube2f* (*Foxp3Cre;Ube2f*^*fl/fl*^) or *Ube2m* (*Foxp3Cre;Ube2m*^*fl/fl*^) or E3s, *Rbx1* (*Foxp3Cre;Rbx1*^*fl/fl*^) or *Sag* (*Foxp3Cre;Sag*^*fl/fl*^).^207–209^ Mice with individual Treg KO of *Ube2f* or *Sag* are healthy and fertile, indicating the neddylation Ube2f-Sag axis is dispensable. However, mice with T cell deficiency of either *Ube2m* or *Rbx1* develop fatal inflammatory disorder with severely impaired the immune suppressive functions, indicating that the Ube2m-Rbx1 axis is essential for the maintenance of Treg cell fitness.^207^,^208^ Double KO of either E2s or E3s showed that both E2s or E3s have functional redundancy in the functional maintenance of Treg cells.^209^

### The role in stem cell maintenance

The neddylation-activated CRLs play critical regulatory role in the maintenance of stem cell homeostasis, self-renewal and differentiation by targeting degradation of several stemness-governing factors, such as c-MYC,^210^ SOX2,^211^ Notch.^212^ The F-box protein FBXW7 is elevated in embryonic stem cells (ESCs) and its proper expression is essential for proliferation and maintenance of ECSs.^213,214^ The SAG-CUL1-FBXW7 E3 ligase regulates the NF1-RAS pathway by targeting NF1 for ubiquitylation and degradation, therefore playing an important role in embryogenesis.^170^ Genetically deletion of *Sag* in mice causes Nf1 accumulation to suppress the Ras pathway, thus inhibiting endothelial differentiation, teratoma proliferation and angiogenesis in ESCs.^170^ *Sag* deletion also impairs the retinoic acids-induced ESCs differentiation.^215^

FBXW7 acts as a key regulator for the differentiation of neural stem cells by targeting Notch family members.^212^ Mice with brain-specific *Fbxw7* deletion show morphological abnormalities of brain and die shortly after birth.^212^ Pathologically, the neural stem cells with *Fbxw7* deletion lose neuronal differentiation, but skew the differentiation toward astrocytes due to the accumulation of Notch1 and Notch3.^212^ Similarly, complete deletion of *Fbxw7* in the hematopoietic compartment contributes to the development of T cell acute lymphoblastic leukemia (T-ALL) by activating the Notch pathway,^216,217^ and increases leukemia-initiating cells (LICs) by causing the accumulation of c-MYC.^218^ In LICs of chronic myeloid leukemia (CML), FBXW7 is required for maintenance of quiescence of LICs by reducing the c-MYC.^219^ Thus, targeting FBXW7 to purge the LICs with the combination of imatinib provides an attractive therapeutic approach for CML therapy.^219^ Hepatic deletion of Fbxw7 results in elevated proliferation of the biliary system in mice, and facilitates the differentiation of liver stem cells to cholangiocyte rather than hepatocyte due to Notch1 accumulation in vitro.^220^ Furthermore, FBXW7 appears to suppress the stemness potential of cholangiocarcinoma cells by targeting mTOR for degradation.^221^

FBXW2, another F-box protein of CRL1, was recently found to promote ubiquitylation and degradation of MSX2 (muscle segment homeobox 2), a transcription repressor that down-regulates SOX2, leading to de-repression of SOX2 expression, and eventually promote breast cancer stem cell property.^211,222,223^ β-TrCP, yet another F-box protein of CRL1, promotes ubiquitylation and degradation of REST, leading to inhibition of differentiation of ESCs into neural stem cells by blocking the REST-initiated expression of neuron-specific genes.^224,225^ Furthermore, CRL2^VHL^ E3 was shown to be critical for the quiescence and long-term maintenance of hematopoietic stem cells (HSCs) by promoting HIF-1α ubiquitylation and degradation.^226^ Biallelic deletion of VHL, which causes the HIF-1α stabilization, maintains cell cycle quiescence in HSCs, while deletion of HIF-1α otherwise causes a loss of quiescence, specifically in the HSC compartment.^226,227^ NANOG is an essential transcriptional factor required for the maintenance of ESCs.^228^ It was found that CRL3^SPOP^ E3 promotes ubiquitylation and degradation of NANOG, thus inhibiting the self-renewal of prostate cancer stem cells.^122^ Likewise, the CRL4^DCAF5^ E3 also regulates the self-renewal and pluripotency of ESCs by promoting ubiquitylation and degradation of SOX2.^229^ Similarly, the CRL4A^DET1-COP1^ E3 also promotes SOX2 ubiquitylation for proteasomal degradation to enhance neural progenitor cell differentiation.^230^ The CRL5^ASB4^ E3 is an oxygen-sensitive ligase that is highly activated during the differentiation of ESCs into endothelial cell lineages.^231^ By targeting ID2 for ubiquitylation and degradation, CRL5^ASB4^ E3 promotes differentiation of trophoblast cells into placental vascular lineages.^232^ All the above pieces of evidence suggest that neddylation pathway, by controlling the activation of CRLs, indirectly regulates stem cell property. Indeed, MLN4924 treatment at nanomolar concentration remarkably stimulates in vitro tumor sphere formation and in vivo wound healing, tumorigenesis and differentiation of human cancer cells and mouse embryonic stem cells, partially through c-MYC accumulation.^233^ It was further demonstrated that PLGA nanoparticles coated with biomimetic macrophage membranes loaded with MLN4924 enhance the healing of diabetic wounds.^234^

### The role in mitochondria function

Mitochondria, the power house of a cell, are essential hubs for bioenergetics, and malfunction of mitochondria is associated with several human diseases, including metabolic disorders and cancers.^235^ Structurally, mitochondrial organelles are highly dynamic by undergoing fusion, fission, transport and degradation, which is crucial for maintaining mitochondrial functions and cellular metabolic reprogramming.^236^ Disrupted mitochondrial dynamics, especially the switch towards fission status, is significantly associated with tumorigenesis^236^ How neddylation pathway regulates the mitochondrial structure and functions has been extensively reviewed recently.^109^ Our previous studies revealed that blockage of neddylation pathway by MLN4924 treatment or NAEβ knockdown altered mitochondrial morphology by triggering the fission-to-fusion conversion, resulting in reduced tricarboxylic acid cycle but increased oxidative phosphorylation. Mechanistically, MFN1, a fusion-promoting protein is a substrate of neddylation-activated E3 CRL1^β-TrCP^. Neddylation blockage inactivates CRL1^β-TrCP^, leading to accumulation of MFN1 to induce the fission-to-fusion conversion.^237^ The neddylation process is also highly activated in hepatic mitochondria, and neddylation of the mitochondrial electron transfer flavoproteins (ETFA and ETFB) caused their stabilization by blocking their ubiquitylation and degradation, eventually facilitating fatty acid β-oxidation.^238^

Reactive oxygen species (ROS) are constantly generated from oxygen during the respiration, and mitochondria are the major place for ROS generation, since it consumes ~80% of oxygen during oxidative phosphorylation.^239^ The homeostasis of ROS (production and scavenge) is important for proper execution of biological processes, and overproduction of ROS contributes to diverse human diseases, including cancer.^240^ Nuclear factor erythroid 2-related factor 2 (NRF2), an antioxidant transcription factor, is known to play a major role in maintaining the redox homeostasis by transcriptionally activating downstream genes encoding various antioxidant proteins in cellular defense against ROS.^241^ NRF2 is also a well characterized substrate of neddylation-activated CRL1 and CRL3 E3s.^21^ When degraded by CRL1 E3, NRF2 is first phosphorylated by glycogen synthase kinase 3, followed by recognized by β-TrCP for targeted ubiuqitylation and degradation. When degraded by CRL3 E3, NRF2, under the normal conditions, is recognized by Kelch-like ECH-associated protein 1 (Keap1), which recruits CRL3 for targeted ubiquitylation and degradation.^21^ Under the oxidative stresses, the cysteine residue on Keap1 is oxidized, which causes its conformation change to inactivate CRL3^Keap1^, resulting in NRF2 accumulation to induce transcriptional expression of phase II antioxidant enzymes for ROS scavenge.^242,243^

### The role in energy metabolism

Energy metabolism is the process of generating ATP from nutrients, including mainly glucose, lipids, and proteins. Neddylation pathway has been implicated in the process of glucose metabolism. Fasting or caloric restriction caused an increased hepatic protein levels of NAE1 and NEDD8, while sugar-fed mice had reduced levels of neddylated cullins. Blockage of hepatic neddylation by MLN4924 or liver-specific knockdown of NAE1 or NEDD8 reduced gluconeogenic capacity and contributed to insulin resistance.^244^ Mechanistically, neddylation of phosphoenolpyruvate carboxykinase 1 (PCK1) at three lysine residues—K278, K342, and K387, increased its gluconeogenic activity by relocating two loops surrounding the catalytic center into an open configuration.^244^ The signaling adaptor sequestosome 1 (SQSTM1)/p62 appears to inhibit the interaction of DCNL1 with cullin2 and abolish the cullin2 neddylation to inactivate CRL2^VHL^ E3 ligase activity, leading to HIF-1α accumulation to promote the Warburg effect by enhancing the glucose uptake and lactate production in renal cell carcinoma.^245^ Likewise, neddylation blockage by MLN4924 also promotes the Warburg effect by activating PKM2 via inducing its tetramerization.^237,246^

Glutamine metabolism is important for energy supply and redox homeostasis, the pleiotropic glutamine also contributes to the maintenance of tumorigenic state.^247^ Our recent study revealed that CRL3^SPOP^ targets ASCT2, a major glutamine transporter, for ubiquitylation and degradation, whereas neddylation inhibition by MLN4924 promotes glutamine uptake by inactivating CRL3^SPOP^ to enhance glutamine uptake. Biologically, an ASCT2 inhibitor that blocks glutamine uptake sensitizes breast cancer cells to MLN4924.^248^

**Fig. 4:**
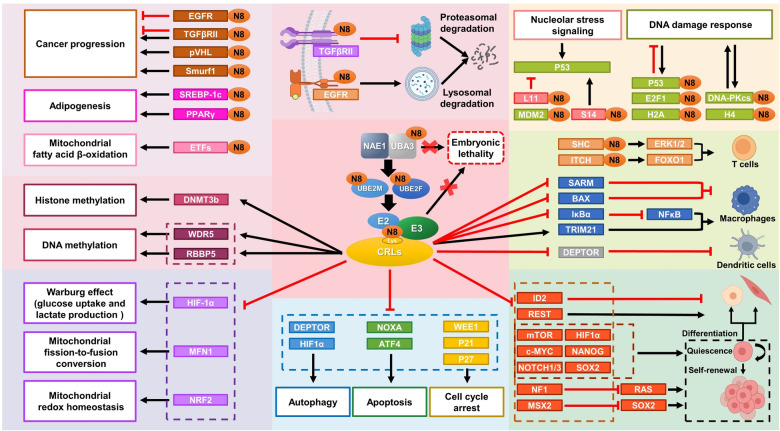
Neddylation regulation of several key biological processes via NEDD8 attachment to various substrates. By attachment of NEDD8 to its cullin and non-cullin substrates, neddylation regulates various key biological processes, including signaling transduction and epigenetic regulation, cellular stress and immune responses, cell growth and organ development, stem cell maintenance, mitochondria function and energy metabolism. Created at BioRender.com

Neddylation pathway is also actively involved in regulation of lipid metabolism. It was observed that NEDD8 is significantly upregulated in white adipose tissues from obese mice.^249^ The PPARγ (peroxisome proliferator-activated receptor gamma), a nuclear hormone receptor driving adipogenesis,^250^ is subject to MDM2-mediated neddylation, leading to its stabilization. Blocking neddylation by NEDD8 knockdown or MLN4924 treatment substantially impairs the adipocyte differentiation and fat tissue development due to downregulation of PPARγ.^251,252^ Sterol regulatory element-binding protein-1c (SREBP1c) is an important regulator for lipid homeostasis by inducing lipogenesis in liver.^253^ Aberrantly activation of SREBP1c links to progression of hepatic steatosis and eventually development of cirrhosis and liver failure.^254^ Furthermore, MDM2 was found to promote the conjugation of NEDD8 to SREBP1c, the neddylated SREBP1c is stabilized due to blockage of its ubiquitylation, consequently driving lipid accumulation in the liver.^255^ Under high glucose conditions, XIAP ligase promotes neddylation of PTEN on Lys197 and Lys402 to induce the nuclear import of PTEN, which dephosphorylated FASN, a critical enzyme in fatty acid synthesis, to inhibit its ubiquitylation and degradation by TRIM21 E3, leading to increase the de novo fatty acid synthesis and tumor progression in breast cancer.^256^ Taken together, neddylation pathways regulate a variety of biochemical and biological processes directly (via NEDD8 or neddylation enzymes) or indirectly (via activation of CRLs or functional modulation of non-cullin substrates) to ensure a healthy operation of a cell (Fig. 4).

## Abnormal neddylation in human diseases

**Fig. 5:**
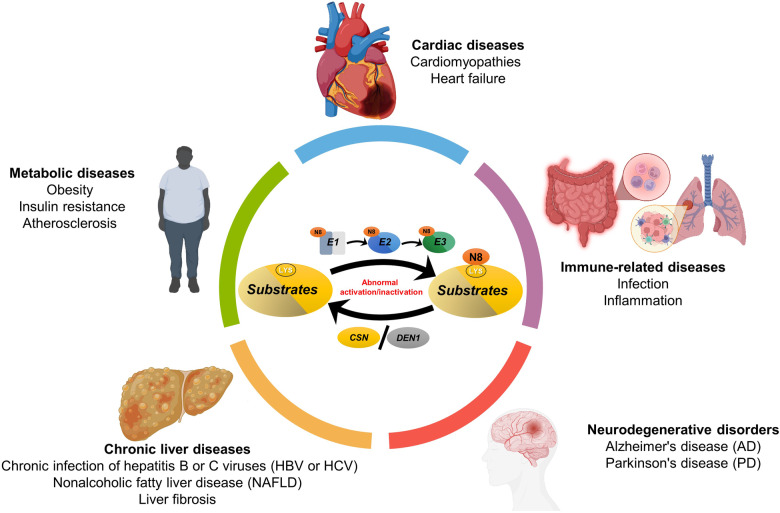
Abnormal neddylation in human diseases. Dysregulation of neddylation or de-neddylation pathways is associated with several human diseases, such as metabolic disorders, liver dysfunction, neurodegenerative disorders, cardiac diseases and immune-related inflammatory and infectious diseases

**Fig. 6:**
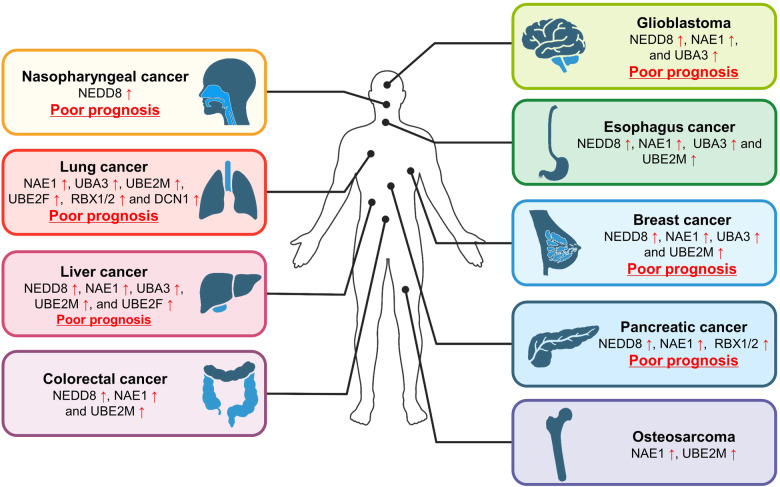
Abnormal neddylation in cancers. Neddylation pathway is hyper-activated in many types of human cancers, and overexpression of some neddylation components is associated with advanced stages of diseases and poor survival of cancer patients. Created at BioRender.com

The neddylation pathway is tightly regulated in order to maintain protein homeostasis and proper operation of various cellular processes. Dysregulation of neddylation or de-neddylation pathways is associated with several human diseases, including metabolic disorders,^251^ liver dysfunction,^238^ neurodegenerative disorders,^165^ cardiac diseases,^257^ immune-related diseases^102^ (Fig. 5), and most importantly, cancer (Fig. 6).^21^ Targeting neddylation is, therefore, considered as a potential therapeutic approach for the treatment of these diseases.

### Metabolic diseases

Metabolic diseases are a group of medical conditions with the clinical manifestation of high glucose, low levels of HDL cholesterol, high levels of LDL and triglycerides in the blood, high blood pressure, large waist circumference or “apple-shaped” body.^258^ Metabolic diseases are caused by errors in metabolism, which include type 2 diabetes (T2D), hypertension, hypertriglyceridemia, atherosclerosis, and among others.^259^ Obesity is one of the signs of metabolic syndrome that significantly increases the risk of related metabolic diseases.^260^ Importantly, the levels of NEDD8, NAE1 and neddylated cullins are significantly higher in the group of people with obesity and type II diabetes.^244,261^ Neddylation-mediated PPARγ stabilization is crucial for adipogenesis and lipid accumulation within adipocytes, and neddylation inhibition by MLN4924 therefore effectively inhibits obesity induced by the high-fat diet.^251^ Our collaborative study recently showed that NEDD8-specific E2 conjugating enzyme, UBE2M, is highly associated with obesity and obesity-related metabolic diseases. Mechanically, UBE2M promotes TRIM21 neddylation to enhance TRIM21-mediated VHL ubiquitylation. As such, HIF-1α, the substrate of CRL2^VHL^, is accumulated to induce IL-1β production from macrophages, thus aggravating obesity-induced inflammation.^187^

Moreover, obesity-related inflammation, insulin resistance, and diabetes are closely related.^262^ Hepatic insulin resistance is highly associated with systemic insulin resistance and dyslipidemia.^263,264^ The nutrient and stress-sensing kinases, upon abnormal activation, phosphorylate insulin receptor substrate (IRS) for proteasome degradation, which is a key underlying mechanism for hepatic insulin resistance. MLN4924 treatment increases hepatic insulin signaling and decreases hepatic glucose production by inhibiting CRL-mediated IRS degradation, indicating that neddylation pathway indeed regulates the insulin resistance.^265^

Atherosclerosis is a chronic inflammatory disease characterized by infiltration of blood vessels by lipids and leukocytes, which is mostly caused by metabolic disorders.^266^ Deneddylase CSN5 was found to be overexpressed in human atherosclerotic lesions,^267^ and mice lacking myeloid Csn5 developed larger atherosclerotic lesions.^183^ Finally, the histone deacetylase 6 (HDAC6) is also involved in modulating atherosclerosis, and inhibition of HDAC6 neddylation significantly increases its activity to protect against endothelial dysfunction and atherosclerosis.^145^

### Chronic liver diseases

Chronic liver disease (CLD) is a continuous process of inflammation, destruction, and regeneration of liver parenchyma, leading to development of fibrosis, cirrhosis and eventually carcinoma.^268^ Dysregulated neddylation pathway was reported in various pathological conditions during the CLD development. For instance, mice with liver-specific knockout of *Nedd8* or *Uba3* died between postnatal days 1 to 7 or 13 to 18, respectively, with spontaneous fatty liver and senescent hepatic cells.^238^ Furthermore, both NAE1 and global levels of neddylated proteins are elevated during the liver fibrosis.^269^ Although the initial triggering events that activates neddylation pathway during CLD remains elusive, diverse stress conditions, including heat shock and oxidative stress, are likely the contributing factors.^15,61^ Interestingly, it was reported that UBE1, an ubiquitin E1, rather than NAE1, promotes the conjugation of NEDD8 to various proteins under the stress circumstances (e.g., heat shock and oxidative stress).^11,15^ This E1 mediated ubiquitin-NEDD8 “cross-talk” certainly provides a remarkable amplification of the NEDD8 proteome,^15,270^ but its physiological and pathological implications remain unknown.

The diversified etiologies of CLD, primarily include chronic infection of hepatitis B or C viruses (HBV or HCV), chronic consumption of alcohol or drugs, chronic aberrant metabolic conditions [e.g., nonalcoholic fatty liver disease (NAFLD)], and abnormal autoimmunity.^271^ Chronic infection of HBV is a primary cause for CLDs, particularly for Chinese patients. HBV-encoded X protein (HBX) is a small regulatory protein that exhibits pleiotropic activities to stimulate virus gene expression and replication, thereby promoting the development of CLD^272^ Notably, the damage-specific DNA binding protein 1 (DDB1), an adaptor protein of CRL4 E3 ligase, is a well-characterized HBX binding partner.^273,274^ HBX redirects CRL4 E3 ligase activity to degrade the negative regulators of HBV transcription, including PRMT1,^275^ WDR77,^276^ and SMC5/6,^277^ thereby promoting HBV gene expression and replication. As such, neddylation pathway activates CRL4 to affect HBV replication indirectly. Moreover, HBX is directly neddylated by HDM2 (human homolog of MDM2), which stabilizes HBX by preventing its ubiquitylation and degradation, thus facilitating the transcription of HBV genes.^278^ Moreover, HBV infection induces NEDD8 expression in human liver cells, whereas NEDD8 knockdown or MLN4924 treatment inhibits HBV replication.^279^ Thus, neddylation pathway is actively involved in HBV-induced CLDs.

NAFLD is characterized by excessive accumulation of triglyceride in liver cells, known as steatosis, which frequently progresses to a more severe stage of nonalcoholic steatohepatitis (NASH), consisting of hepatic steatosis, inflammation, and fibrosis.^280^ Neddylation was reported to regulate this process. Specifically, the mitochondrial electron transfer flavoproteins (ETFA and ETFB) are stabilized by neddylation via reducing their ubiquitylation, eventually preventing fasting-induced liver steatosis.^238^ On the other hand, neddylation blockade inhibits the accumulation of lipid droplets, in part, via increasing mitochondrial fatty acid oxidation (FAO), which maintains liver function and attenuates pathological remodeling of liver structure, thus delaying the development of obesity and HFD-induced NAFLD.^252^ Furthermore, the serine-rich splicing factor 3 (SRSF3) is essential to hepatocyte differentiation, and liver glucose and lipid metabolisms.^281^ Genetic depletion of SRSF3 in hepatocytes causes fibrosis, steatohepatitis.^282^ Notably, SRSF3 protein is neddylated at lysine 11, which enhances its proteasome degradation via an undefined mechanism, thereby contributing to the progression to NASH and cirrhosis.^282,283^

Liver fibrosis is a common consequence of most chronic inflammatory diseases, including viral infection, alcohol, and NASH.^271^ Notably, altered neddylation pathway is associated with liver fibrosis, since the levels of NAE1 and neddylated cullins are increased during liver fibrosis, originating from both hepatitis B infection and alcohol abuse.^269^ Mechanically, neddylation activation of CRL1^βTrCP^ ubiquitylates IκBα for proteasome degradation, resulting in p65 nuclear localization to activate NF-κB, leading to enhanced expression of inflammatory cytokines to promote the inflammation and fibrogenesis.^284^ Moreover, neddylation modification stabilizes TGFβ-RII to activates the TGFβ signaling, which plays an important role in activation of hepatic stellate cells (HSCs) and liver fibrosis.^269,285^ Finally, neddylation of EphB1 (Erythropoietin-producing human hepatocellular receptor tyrosine kinase B1) enhances its kinase activity, and significantly increases the number of HSCs, therefore contributing to liver fibrosis induced by CCl4 in mice.^286^ Thus, targeting neddylation pathway appears to be a potential and novel therapeutic approach for the treatment of liver fibrosis and CLD.^269,287^

### Neurodegenerative disorders

Neurodegenerative diseases are a group of disorders characterized by progressive loss of selectively vulnerable populations of neurons, including Alzheimer’s disease (AD), Parkinson’s disease (PD), Huntington’s disease. The homeostasis of neddylation is important for the nerve growth, synapse strength, neurotransmission and synaptic plasticity, and dysregulated neddylation pathway is associated with and may be related causally to the development of neurodegenerative diseases.^288^ Abnormal deposition of amyloid protein Aβ and the formation of intracellular neurofibrillary tangles by tau hyperphosphorylation are the two major pathological features of AD.^289^ The UPS plays an essential role in the pathogenesis and progression of AD by controlling the accumulation of insoluble proteins in the brain, implying that neddylation pathway via modifying various E3 ligases, may also be involved in AD pathogenesis.^290^ More directly, dysregulated neddylation pathway has been found in AD. For examples, while NEDD8 is normally localized in the nucleus of normal hippocampal pyramidal cells and granule cells, it is mostly located in the cytoplasm of AD hippocampal neurons.^291,292^ Furthermore, APP (β-amyloid precursor protein) is the precursor of Aβ, which is generated from APP by sequential cleavages by β-secretase and γ-secretase complex. Notably, APP-binding protein 1 (APP-BP1), also known as NAE1, acting as a regulatory subunit to complex with catalytic subunit of UBA3, to form an active NEDD8-activating enzyme, which neddylates CUL1 to activate CRL1/SCF for the proper processing of APP.^9,291^ Finally, presenilin (PS), the constituent of γ-secretase, is the substrate of neddylation-activated CRL1, and PS ubiquitylation could alter cellular levels of unprocessed PS and increase in the production of Aβ.^293^

PD is another well-known neurodegenerative disease featured by the progressive degeneration of dopaminergic neurons in the substantia nigra complex with Lewy bodies presented in the lesion tissues.^294^ Several gene mutations, including PARKIN and PINK1, are responsible for the pathogenesis of PD.^295^ Interestingly, PARKIN and PINK1 are the substrates of neddylation modification, and neddylation potentiates the PARKIN E3 ligase activity and increases the stability of a 55 kDa PINK1 proteolytic fragment, which is an active PINK1 form.^296^ Dysregulated neddylation of PARKIN and PINK1 likely contributes to PD pathogenesis.^297,298^

### Cardiac diseases

Heart disease is one of the leading causes of global death.^299^ Recently, substantial pieces of evidence supported the notion that abnormal homeostasis of cellular proteins a key mechanism for the initiation and development of cardiac diseases.^257^ It was reported that the levels of both neddylation enzymes and neddylated proteins are high in embryonic hearts, but significantly reduced at 1 week after birth.^7,167^ Transient MLN4924 treatment of neonatal rats inhibits cardiomyocyte proliferation and cardiac hypertrophy, and improves cardiac function, supporting the notion that neddylation play an important role in perinatal cardiac growth.^300^ Interestingly, mice lacking *Nae1* show proliferation arrest of cardiomyocytes at E14.5 and pronounced ventricular non-compaction by E16.5, ultimately leading to heart failure and neonatal lethality.^167^ On the other hand, the neddylated proteins are significantly accumulated in failing hearts from patients with dilated and ischemic cardiomyopathy, suggesting that dysregulated neddylation is indeed involved in cardiomyopathies.^257^

Mice with cardiac-specific deletion of *Csn8* have increased total neddylated proteins and develop cardiac hypertrophy, dilated cardiomyopathy and eventually died from heart failure by age of 4 weeks.^301^ Furthermore, tamoxifen-induced *Csn8* knockout in the adult heart induces rapid heart failure and mice die within 2 weeks after induction.^302^ Thus, the CSN8-mediated deneddylation is required in the maintenance of cardiac integrity in both postnatal and adult hearts. Mechanistically, loss of CSN8 is likely to activate the RIPK1-RIPK3 pathway to induce massive cardiomyocyte necroptosis, therefore contributing to heart failure.^301,303^ As such, treatment of the RIP1K inhibitor or deletion of even one allele of Ripk3 inhibited the death of cardiomyocytes and prolonged the lifespan of *Csn8*-deficient mice.^304^ Thus, a precise balance between neddylation and deneddylation is the key to maintain the normal heart physiology.^257^

### Immune-related diseases

Neddylation becomes a critical regulatory mechanism for both innate and adaptive immune system, and their associated signal pathways.^305^ Dysregulation of neddylation by overactivating CRLs affects the functions of immune cells by enhanced degradation of key signal proteins involved in immunoregulation, thus influencing various immune-related diseases, including viral infection, inflammation, and autoimmune diseases.^305,306^

By promoting neddylation of proteins involved in the host restriction factors (a group of cellular proteins that inhibit the replication of viruses), neddylation pathway has been shown to modulate the process of viral infections.^307^ During virus infection, the virus proteins, such as Vif, hijacks CRL5 to promote ubiquitylation and degradation of host restriction factors/anti-viral proteins APOBECs (for review, see^308^). The blockage of neddylation by MLN4924 represses the degradation of host restriction factors, APOBEC3G and SAMHD1, therefore restoring the restriction on human immunodeficiency virus (HIV).^309^ At the early phase of infection by herpes simplex virus type I (HSV-1), neddylation is required for production of type I interferon as the host innate immune response. This process was inhibited by MLN4924 or UBA3 knockdown via inactivation of NFκB through induced IκBα accumulation.^310^ Similarly, etoposide, a neddylation inducer, decreases lung lesions in a mouse model of SARS-CoV-2 infection by activating CRL4B^PRPF19^ to degrade ORF6, an accessory protein of SARS-CoV-2, which significantly inhibits the production of interferon.^311^ Other examples include neddylation of the polymerase basic protein 2 (PB2) of influenza A virus (IAV) by HDM2 causes its destabilization, thus blocking the replication of influenza A virus,^312^ and neddylation of VP2, the structural protein of enterovirus 71 (EV71) on K69 residue promotes its degradation to suppress multiplication of the virus.^313^

For inflammation responses triggered by a variety of stimuli, neddylation pathway also plays an essential role by regulating the production of proinflammatory cytokines mainly via controlling the CRL1/NFκB signal. Inactivation of neddylation causes accumulation of IκB to inactivate NFκB and inhibits the expression of proinflammatory cytokines (eg, TNFα and IL6), thereby alleviating LPS-induced inflammation.^113,314^ Similarly, neddylation immunomodulation has also been described in mucosal inflammation, MLN4924 enhances the barrier function of intestinal epithelial cells through a CRL2/HIF-1α dependent mechanism,^121^ with potential to alleviate inflammatory bowel diseases. Finally, neddylation regulation of other inflammatory diseases as well as several autoimmune diseases have been extensively reviewed most recently,^305^ highly suggesting that neddylation pathway is a promising therapeutic target for the treatment of these diseases.

### Cancers

Accumulated lines of evidence have clearly demonstrated that neddylation pathway is significantly overactivated in a number of human cancers, and in most cases, its overactivation correlates with worse survival of cancer patients^315^ (Fig. 6).

In lung cancers, including large cell neuroendocrine lung carcinoma (LCNEC), lung adenocarcinoma (LUAD) and lung squamous cell carcinoma (LUSC), NEDD8-related proteins are found to be dysregulated and associated with malignant stages.^316^ Compared to lung tissues, the mRNA expression of UBA3, NAE1 and UBE2M were significantly higher in LUAD, LUSC and LCNEC tissues, and higher UBE2M mRNA is correlated with poor differentiation in the LUAD.^316–318^ Moreover, immuno-histochemical staining and western blot analyses showed that the levels of NAE1, UBA3 UBE2M, UBE2F, RBX1/2 and DCN1 are much higher in tumor tissues than adjacent normal tissues.^317,319–321^ Furthermore, the elevated global neddylation is associated with unfavorable overall survival of lung cancer patients.^316,317,322^ Mechanically, overactivated neddylation pathway appears to activate CRLs to ubiquitylate and degrade tumor suppressors (e.g., p21 and p27), thus facilitating lung cancer progression.^316,317^ Furthermore, neddylation pathway promotes recruitment and infiltration of myeloid-derived suppressor cells (MDSCs) to lung tumor sites via the NF-κB-mCXCL5 axis, therefore contributing to tumor progression.^323^ Our previous study revealed that the UBE2F-SAG pair neddylates CUL5 to activate CRL5 for targeted ubiquitylation and degradation of proapototic protein NOXA, leading to enhanced survival of lung cancer cells.^324^ UBA3 upregulation in lung cancer cells promotes p53 neddylation to inhibit its transcriptional activity, therefore facilitating cell proliferation and invasion.^318^ Collectively, activation of neddylation pathway serves as an oncogenic signal.

In breast cancer, the levels of NEDD8, neddylation E1 NAE1 and UBA3, E2 UBE2M, and E3 XIAP are all significantly upregulated, compared to matched adjacent normal tissues, which positively correlated with the worse patient survival.^131,256,325,326^ The hyperactivated neddylation pathway facilitates HER2 neddylation to inhibit its ubiquitylation and subsequent degradation, therefore promoting the progression of HER2-positive breast cancer.^131^ Neddylation pathway also regulates estrogen-related receptors (ERRs) by inducing CRL1-mediated ubiquitylation and degradation of ERRs, eventually leading to breast cancer progression.^327^ Notably, Ran-binding protein 2 (RanBP2) was identified as a neddylation E3, which neddylates MKK7 (mitogen-activated protein kinase kinase 7) to inhibit its basal kinase activity. RanBP2 knockdown reduces proliferation and impairs EMT phenotype, which is rescued by simultaneous MKK7 knockdown in human breast cancer cells.^328^ The tumor suppressor PTEN is subjected to neddylation, triggered by high levels of glucose. Interestingly, neddylated PTEN accumulates predominantly in the nucleus to promote proliferation, metabolism and progression in breast cancer.^256^ Finally, we found that neddylation pathway controls glutamine uptake and metabolism. Mechanistically, neddylation inhibition by MLN4924 or UBA3 knockdown inactivates CRL3^SPOP^ E3 ligase to cause accumulation of glutamine transporter ASCT2 for enhanced glutamine transports and metabolism in breast cancer cells.^248^

In colorectal cancer, the levels of NEDD8, NAE1 and UBE2M are all remarkably elevated in colorectal cancer tissues, as compared to matched adjacent normal tissues.^46^ Notably, a C2-WW-HECT ligase, Smurf1, is positively correlated with NEDD8, NAE1 and UBE2M. Mechanistic study revealed that Smurf1 acts as a NEDD8 ligase to promote its auto-neddylation when coupling with NEDD8 and UBE2M, which in turn enhances Smurf1-mediated ubiquitylation of Smad4, Smad5, RhoA or ING2, leading to accelerated colorectal cancer progression.^46^ Furthermore, Smurf1, acting as neddylation E3, promotes RRP9 (ribosomal RNA processing 9, U3 small nucleolar RNA binding protein) neddylation to induce pre-rRNA processing, also accelerating the proliferation of colon cancer cells.^329^ Oncoprotein Hu antigen R (HuR) is subjected to neddylation by MDM2, leading to its stabilization. High levels of HuR correlates with tumor malignancy and colon cancer metastases.^330^ Finally, SHP2 (Src homology region 2–containing protein tyrosine phosphatase 2) is also subjected to neddylation, leading to its inactivation through a conformation change, which regulates macrophage-mediated engulfment of opsonized colorectal tumor cells.^331^

In human intrahepatic cholangiocarcinoma, the levels of NEDD8, NAE1, UBA3, and UBE2M are all elevated and apparently correlated with worse clinical characteristics, while only NAE1 is the independent factor associated with postoperative recurrence.^332^ In hepatocellular carcinoma (HCC), the entire neddylation pathway, including NEDD8, E1 (NAE1 and UBA3), E2 (UBE2M and UBE2F), E3 NEDD8-ligases (RBX1, RNF7/RBX2/SAG and MDM2), and deneddylation enzymes (COPS5, UCHL1 and USP21) is over-activated, and upregulated NAE1, UBE2M, and UCHL1 is associated with poor survival of HCC patients.^325,333,334^ Mechanistic studies showed that upon activation of neddylation pathway, AKT and LKB1, hallmarks of proliferative metabolism, are neddylation and stabilized, which is related to the development of liver cancer.^333^ The tumor suppressor, RhoB (a Rho family member of small GTPases) was identified as the substrate of the neddylation-CRL pathway. Neddylation-activated CRL2 E3 ligase targets RhoB for ubiquitylation and degradation, as a key molecular event that drives liver carcinogenesis.^335^ The master lipogenic transcription factor SREBP-1 is also subjected to UBE2M-mediated neddylation, and SREBP-1 neddylation leads to its stabilization, which is related to progression of liver cancer.^325^ Likewise, the oncoprotein Hu antigen R (HuR) is stabilized by Mdm2-mediated neddylation, which contributes to liver cancer metastases.^330^

In human esophageal squamous cell carcinoma, the levels of NEDD8, NAE1, UBA3, and UBE2M are increased significantly, which is positively correlated with high-grade malignancy and postoperative recurrence as well as worse overall survival of patients.^336,337^ The hyperactivated neddylation pathway activates CRL/SCF^βTrCP^ E3 to destabilize the activating transcription factor 4 (ATF4), thus protecting cells from apoptosis by inhibiting the extrinsic apoptosis mediated by the ATF4–CHOP–DR5 axis, leading to cancer cell survival.^337^

In glioblastoma, the levels of NAE1, UBA3 and UBE2M are increased significantly with positively correlation of high-grade malignancy and postoperative recurrence as well as worse overall survival of patients.^338^ The protein levels of UBE2M, RBX1, and SAG/RBX2 are elevated in pancreatic cancer tissues, and NAE1 and UBE2M are higher in osteosarcoma tissues than adjacent normal tissues.^339–341^ The elevated NEDD8 expression in nasopharyngeal carcinoma is correlated with positivity of lymph node metastasis, shorter disease-free survival and poor overall patient survival.^342^

Despite the large amount of clinical data have demonstrated that neddylation-associated proteins are clearly upregulated in tumor tissues, it is still not clear whether activated neddylation pathway plays a causal or a consequential role during tumorigenesis. To this end, we generated conditional *Sag* or *Rbx1* KO models in lung tissues, and found that deletion of either *Sag* or *Rbx1* remarkably suppressed lung tumor formation induced by *Kras*^*G12D*^ via inactivating CRLs,^343,344^ indicating that neddylation E3s Sag and Rbx1 are promoting factors in *Kras*^*G12D*^-induced lung tumorigenesis. Nevertheless, more conditional KO mouse models are needed for neddylation-pathway genes to determine their causal role during tumorigenesis, induced by activated oncogenes (e.g., Kras, or Wnt/β-catenin) or deletion of tumor suppressors (e.g., p53 or Pten).

## Targeting neddylation for anticancer therapy

**Fig. 7:**
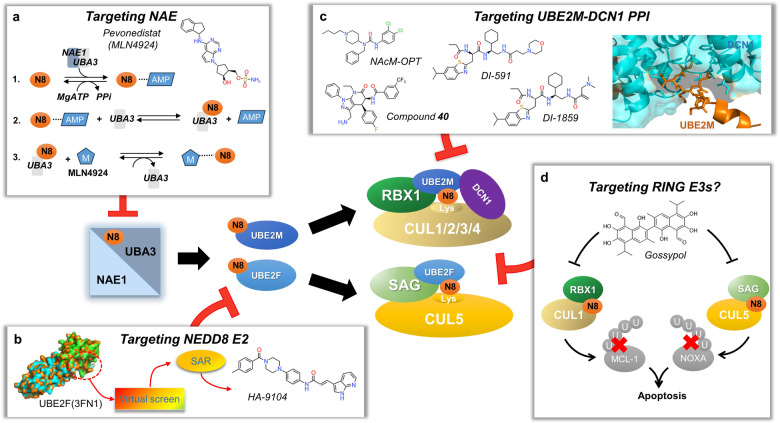
Small molecular inhibitors targeting neddylation pathway. **a** E1 inhibitor, represented by MLN4924. **b** UBE2F E2 inhibitor HA-9104. **c** Several inhibitors that disrupt the UBE2M-DCN1 binding (PDB ID: 3TDU), represented by NAcM-OPT, compound 40, DI-591 and DI-1895. **d** Non-specific E3 inhibitor of gossypol targeting CUL1-RBX1 and CUL5-RBX2. Created at BioRender.com

Over-activation of neddylation pathway in variety of human cancer, along with its positive correlation with poor survival of cancer patients, highly suggests that this pathway is an promising drug target for pan cancers. Indeed, many preclinical studies, using the approaches including those in molecular and cellular biology, tumor biology, genetically modified mouse models, have validated that is the case,^12,21^ which leads to the advancement of MLN4924 (pevonedistat), the first-in-class neddylation E1 inhibitors, to several phases of clinical trials.^18^ The topic of targeting neddylation for anticancer therapy has been extensively reviewed by a number of investigators including ourselves.^90,345,346^ We will briefly review a few representative well-characterized neddylation inhibitors, which is summarized in Fig. 7.

### NAE E1 inhibitor

MLN4924, a highly selective and first-in-class small molecule inhibitor of neddylation E1 NAE, was first reported in 2009.^18^ By binding to the active site of NAE and form a steady-state covalent adduct with NEDD8 to impair the process of enzymatic reaction,^347^ MLN4924 blocks the entire neddylation reaction. As a result, neddylation of both cullins and non-cullin substrates is inhibited, resulting in inactivation of all CRLs and accumulation of various CRLs substrates. MLN4924 suppresses the growth and survival of many cancer cell lines by inducing cell cycle arrest, apoptosis, autophagy and senescence.^18,122,347,348^ A large number of preclinical studies demonstrate that MLN4924 has an effective anticancer activity for a variety of human cancer cells with tolerable cytotoxicity in mice.^349–353^ More significantly, a series of phase I/II clinical trials were conducted to evaluate its antitumor activity and safety in patients with hematologic or solid malignancies.^354–359^ MLN4924, known as Pevonedistat in clinical trials, has shown some efficacy to cause stable disease, partial response, and in few cases, complete response.^12,21^ More importantly, the FDA has granted a Breakthrough Therapy Designation to the investigational agent MLN4924 for the treatment of patients with higher-risk myelodysplastic syndrome.^360^

### The mechanisms of MLN4924 action

#### Inducing cell cycle arrest

Cell cycle arrest is one type of mechanism by which many anti-cancer agents inhibit the proliferation of cancer cells.^361^ MLN4924 has shown to induces arrest in different phases of cell cycle by causing the accumulation of a variety of CRLs substrates in a manner dependent of cell types. Specifically, in mantle cell lymphoma and activated B cell-like (ABC) diffuse large B-cell lymphoma (DLBCL) cells, MLN4924 treatment causes rapid accumulation of IκBα to inhibit NF-κB, which is important for the G0/G1-to-S-phase transition,^362^ therefore inducing G1 phase arrest and ultimately leading to cell apoptosis.^350,363^ In germinal-center B cell-like (GCB) DLBCL and colon cancer cells, MLN4924 treatment causes the accumulation of CRLs substrate, CDT1 (Chromatin licensing and DNA replication factor 1) via inactivation of CRL1 or CRL4^364^ to deregulate the component of prereplication complex, which enhances DNA synthesis and triggers DNA replication stress to arrest cancer cells in S phase, and subsequent DNA damage and cell death.^18,350,365^ Finally, the most frequent cell cycle arrest induced by MLN4924 is at the G2 or G2/M phase by inactivation of CRL1 to cause the accumulation of Wee1, an inhibitor of G2–M phase transition.^366^ By doing so, MLN4924 induces cell cycle arrest at the G2 or G2/M phase in many types of cancer cells, including live cancer,^348^ cholangiocarcinoma,^332^ glioblastoma,^338^ nasopharyngeal carcinoma,^342^ gastric cancer,^367^ T-cell acute lymphoblastic leukemia,^368^ pancreatic cancer,^369,370^ renal cancer,^371^ urothelial carcinoma,^372^ and Ewing sarcoma.^373^ Interestingly, MLN4924 induces the arrest at the different phase of cell cycle in different types of RB (retinoblastoma) tumor cells. For example, MLN4924 mainly causes G1 arrest in RB1021 cells, but S + G2/M arrest in RB3823 cells, while RB247, WERI-RB1 and Y79 cells appears to undergo G2/M arrest upon MLN4924 treatment. The exact underlying mechanism for these distinct effects is unclear, but it is likely associated with differential modulation by MLN4924 of multiple CRL targets and/or other neddylated proteins in a cell line dependent manner.^352^

#### Inducing apoptosis

Virtually all mammal cells have the ability to self-destruct and maintain homeostasis by undergoing apoptosis.^374^ Induction of apoptosis is the most common effect of MLN4924 contributing to its killing of cancer cells. By causing accumulation of various CRL substrates, MLN4924 triggers apoptosis through multiple mechanisms. By inactivation of CRL1^SKP2^ and CRL4^CDT2^, MLN4924 causes the accumulation of their substrate CDT1 to induce DNA re-replication and subsequent cell apoptosis in HCT-116 cells.^365^ By inactivating CRL1^β-TrCP^, IκBα is accumulated to block NF-κB activation, leading to apoptosis induction in ABC lymphoma and AML cells.^349,350,375^ Also in AML cells, inactivation of NF-κB transcriptionally downregulates miR-155 via decreasing NF-κB and binding to the miR-155 promoter. As a result, SHIP1 (SH2 domain–containing inositol phosphatase), a miR-155 inhibitory target, is increased to inhibit the PI3K/AKT pathway, eventually resulting in cell apoptosis.^376^ Moreover, by inactivating SAG-CRL5, MLN4924 directly causes the accumulation of the proapoptotic protein NOXA,^324,377^ and by inactivating RBX1-CRL1, MLN4924 increases the levels of c-MYC and transcription factor 4 (ATF4) to promote NOXA transcription, both triggering cell apoptosis.^337,378^ Furthermore, ATF4 accumulation also transactivates the transcription factor CHOP to induce the expression of Death Receptor 5 (DR5), which activates caspase-8 to trigger extrinsic apoptosis pathway in esophageal cancer cells.^337^

#### Inducing senescence

Senescence is characterized by enlarged and flattened cellular morphology and positive staining of senescence associated β-galactosidase, which is another important mechanism by which MLN4924 inhibits the growth of cancer cells.^379,380^ The p53/p21 and p16/pRB initiated pathways are the two major axes contributing to senescence induction in response to various stresses.^381^ Indeed, MLN4924 inactivation of CRL1^SKP2^ causes p21 accumulation to induce senescence in a manner independent of the pRB/p16 and p53, thus suppressing the growth of a variety of cancer cells,^379,380^ including osteosarcoma,^340^ lymphoma,^382^ multiple myeloma,^383^ breast cancer,^384^ pancreatic cancer,^369^ and gastric cancer.^367^ Notably, the low dose of MLN4924 is able to induce the senescence irreversibly, suggesting an effective anti-cancer strategy in specific types of cancer.^379,380^

#### Inducing autophagy

Autophagy is a pro-survival process that allows stressed cells to recoup essential building blocks and ATPs for biosynthesis in most cases, which is beneficial to cell survival and chemoresistance.^385^ Upon MLN4924 exposure, a broad type of cancer cells undergo autophagy as a cellular response to protect themselves from senescence and apoptosis.^348,386,387^ Structurally, MLN4924 treatment causes the accumulation of LC3 II, formation of acidic vesicular organelle (AVO), and double-membraned autophagosome.^348^ Mechanistically, MLN4924, on one hand, inhibits mTORC1 activity by inactivating CRL1^β-TrCP^ to cause accumulation of DPETOR,^195,388,389^ a naturally occurring inhibition of mTORC,^390^ and on the other hand, activates the HIF1α-REDD1-TSC1 axis by inactivating CRL2^VHL^-mediated HIF1α degradation to induce autophagy in multiple human cancer cells.^386^ Moreover, given the ROS stress serves as a potential trigger of autophagy,^391^ and impaired mitochondrial electron transport chain serves as a major source of ROS generation.^392^ MLN4924 appears to induce autophagy by enhancing mitochondrial membrane depolarization to induce the generation of ROS,^393^ which is blocked by N-acetyl cysteine (NAC), a classical ROS scavenger, in Huh-7 and Hep G2 cells.^348^ While MLN4924 suppresses the generation of ROS in macrophages by inhibiting the NEDD8-Cullin3-Nrf2 axis,^394^ indicating a cell type-dependent way of ROS induction by MLN4924. Since the autophagy response triggered by MLN4924 is likely a pro-survival signal, the combination of MLN4924 with autophagy inhibitor chloroquine indeed enhances anticancer efficacy by inducing apoptosis,^348,386,393,395^ suggesting a rational drug combination for effective cancer cell killing.

#### Inhibiting angiogenesis

Abnormal angiogenesis is characterized as an important feature of malignant tumors, and disrupting angiogenesis serves as an attractive strategy for anti-cancer therapy.^396^ It was reported that all neddylation enzymes, including NAE1 and UBA3, UBE2M, RBX1, SAG and DCN1 are expressed and functional in HUVECs (human umbilical vein endothelial cells).^121,317^ Neddylation inhibitor MLN4924 apparently inhibits the formation of capillary-like tube networks of HUVECs, and also markedly suppresses angiogenesis and growth in an orthotopic in vivo model of pancreatic cancer.^173,397^ Mechanistic studies reveal that RhoA, a substrates of CRL3 that controls the assembly of contractile actin and myosin filaments structure,^398^ is accumulated upon MLN4924 treatment in HUVECs, and RhoA knockdown rescues MLN4924-induced phenotype, indicating its causal role.^397^ On the other hand, the prolonged exposure of MLN4924 promotes cell cycle arrest and apoptosis in HUVECs by causing the accumulation of p21, p27 and WEE1 and pro-apoptotic proteins NOXA.^173,397,399^ Moreover, MLN4924 was reported to reduce the tumor microvascular density in a liver metastasis mouse model of uveal melanoma by decreasing the VEGF-C expression due to the inactivation of the NFκB signal.^400^ Taken together, it appears that targeting neddylation by MLN4924 or other newly developed small molecules may serve as a novel class of antiangiogenic agents.

#### Inhibiting the survival of cancer stem cells

Cancer stem cells (CSCs) are a small subpopulation of cancer cells with properties of self-renewal and multi-lineage differentiation that are responsible for tumor progression, drug resistance and recurrence.^401^ Abnormal activation of neddylation is implicated in the self-renewal and survival of CSCs, and targeting NAE by MLN4924 thus could become an effective approach in the elimination of CSCs.^200,233,402,403^ Indeed, MLN4924 was shown to have more cytotoxic effect against AML stem and progenitor cells than cytarabine, a standard-of-care agent.^403^ Likewise, the patient-derived glioblastoma stem cells exhibit highly susceptible to MLN4924 through inhibiting the ERK and AKT signaling, while normal human astrocytes are rather resistant.^404^ In CML, NAE1 expression is remarkably increased in CSCs relative to counterparts, and MLN4924 apparently reduces self-renewal and survival of leukemia stem cells via causing the accumulation of p27.^402^ Similarly, MLN4924 suppresses the proliferation and stemness characteristics in nasopharyngeal carcinoma cells.^342^ In breast cancer cells, MLN4924 causes the accumulation of transcription repressor MSX2 by inactivating CRL1^FBXW2^ E3 ligase. As such, the stem cell factor SOX2 is transcriptionally inhibited by increased MSX2, leading to suppression of the stem cell property.^211^ In uveal melanoma (UM), MLN4924 treatment inhibits the self-renewal of spherogenic UM cells in vitro and reduces the frequency of UM CSCs by 98% in vivo through increasing the level of FBXO11 (by an unknown mechanism), which promotes Slug degradation.^400^ Interestingly, our previously study revealed that MLN4924, when applied at nanomolar concentrations, stimulates unexpectedly cell growth and stem cell self-renewal by inhibiting c-MYC degradation and activating EGFR pathways.^233^ Taken together, it appears that inhibition of CSCs survival is another important anticancer mechanism of MLN4924 action when applied at appropriate concentrations, while neddylation blockage with low concentrations of MLN4924 may be used for stem cell therapy and tissue regeneration.

#### Regulating inflammatory responses

Chronic inflammation is a promoting event in the progression and development of some types of human cancer, and targeting inflammatory responses appears to be another promising approach for anti-cancer strategy^405^ Neddylation pathway is implicated to regulate the survival and activity of several immune cells during inflammatory responses, and blocking neddylation pathway by MLN4924 therefore has the robust anti-inflammatory effect under numerous situations.^12,22,185^

In case of macrophages, short-term MLN4924 treatment (12 h) significantly suppresses the proinflammatory cytokine production induced by LPS in macrophages via blocking the NF-κB activity, without affecting cell viability, while prolonged treatment (over 48 h) apparently induces G2 arrest and apoptosis.^185,186^ Consistently, MLN4924 treatment dramatically decreases LPS-induced iNOS expression and NO production, as well as reduces LPS-induced expression of cytokines and chemokines, including IL-1β, TNFα and MCP-1, via inhibiting NF-κB/MAPK-mediated signaling in macrophages.^406^ Moreover, MLN4924-treated macrophages express lower levels of classical cytokines of M1 type such as TNF-α, IL-12, and IL-6; whereas MLN4924 treatment of BMDMs isolated from Apoe^−/−^ mouse (a model for studying cardiovascular disease, atherosclerosis and fat metabolism), shows upregulation of M2-polarizing cytokine IL-13 and the M2 marker Arginase-1, and downregulation of M1 cytokines after 24 h of LPS exposure, suggesting that MLN4924 is likely to skew the macrophage polarization toward an anti-inflammatory M2 status.^183^

In case of T cells, MLN4924 treatment significantly decreases the proliferation, activation, and effector cytokine release of T cells after stimulation with α-CD3 and α-CD28.^201^ Besides, MLN4924 impairs the functions of T cells by inhibiting T-cell receptor/CD28-induced proliferation and cytokine production, through inhibiting the Shc and Erk signaling.^132^ MLN4924 also impedes downstream TCR signaling via interference with the NF-κB pathway, therefore reducing T-cell activation with reduced IL-2 secretion and cell proliferation.^407^ By inhibiting the NF-κB activation, MLN4924 treatment in CD4 + T cells also reduces the differentiation of inducible regulatory T-cells (iTregs), and shifts the polarization in favor of the Th1 phenotype, likely due to the downregulation of the IL-2/STAT5 signaling, a crucial pathway in Treg differentiation, and decreased production of TNF-α, an NF-κB-regulated cytokine.^407^ Similarly, in DC cells, MLN4924 significantly attenuates the LPS-induced TNF-α and IL-6 production by inactivation of NF-κB, thus suppressing the ability of DC to stimulate T-cell response.^191^ Finally, in human microvascular endothelial cells, MLN4924 significantly abrogates the NF-κB pathway and stabilizes the HIF-1α, thus inhibiting the secretion of TNF-α-elicited pro-inflammatory cytokines during the vascular inflammatory response.^175^ Collectively, it appears that MLN4924 exerts promising anti-inflammatory effect by inhibiting the functions of multiple immune cells. Future study should be geared to investigate the in vivo role of MLN4924 in modulating the function of immune cells infiltrated into the tumor microenvironment.

#### Serving as a sensitizer for other anticancer therapies

In addition to acting as a single anticancer agent, MLN4924 has been used in combinations with chemotherapy, radiotherapy and immunotherapy, and demonstrates its sensitizing effect.^12,408^

For chemotherapy, MLN4924 has been shown to sensitize a number of anti-cancer drugs.^409^ For example, MLN4924 sensitizes ovarian cancer cells to cisplatin even in cisplatin-resistant ovarian cancer cells by increasing the levels of pro-apoptotic protein, BIK and inducing DNA damage and oxidative stress both in cell culture model and in mice bearing ovarian tumor xenografts.^410^ Likewise, MLN4924 sensitizing cytotoxicity effect of cisplatin was also seen in human cervical carcinoma^411^ and urothelial carcinoma.^412^ In pancreatic cancer, MLN4924 sensitizes the effect of gemcitabine by inhibiting cell proliferation and promoting cell death via inducing the accumulation of ERBIN, an inhibitor of the RAS-MAPK pathway, and NOXA.^369^ In colorectal cancer, MLN4924 sensitizes topoisomerase I inhibitors via inactivating the DCAF13-CRL4 E3 ligase.^413^ In multiple myeloma, MLN4924 induces REDD1 (regulated in development and DNA damage responses 1) expression at both RNA and protein levels to suppress the AKT and mTOR signaling, thereby enhancing the bortezomib-induced cytotoxicity.^414^ By inducing the accumulation of c-Jun and NOXA, MLN4924 also sensitizes leukemia cells to retinoic acid-induced apoptosis.^215^ In acute myelogenous leukemia (AML)/myelodysplastic syndrome (MDS), MLN4924 combination with Belinostat, a HDAC inhibitor, was shown to trigger more robust DNA damage and apoptosis, resulting in enhanced suppression of tumor growth with longer animal survival in AML xenograft models.^415^ Finally, MLN4924 blocks the FA pathway by inhibiting FANCD2 monoubiquitylation and CHK1 phosphorylation to increase cellular sensitivity to several DNA interstrand crosslinks (ICLs)-inducing agents, such as cisplatin, mitomycin C and hydroxyureain in various cancer cell lines.^416^

MLN4924 also sensitizes various cancer cell lines to targeted therapeutic agents. In breast cancer cells, the AKT signaling pathway is activated to protect cells from apoptosis upon MLN4924 treatment. The combination of MLN4924 with AKT inhibitor MK-2206 causes much greater induction of apoptosis to kill.^417^ Also in breast cancer cells, MLN4924 transcriptionally blocks ER-α expression through SGK1-dependent cytoplasmic localization of FOXO3a. Thus, MLN4924 combination with fulvestrant, an estrogen receptor antagonist, causes maximal growth suppression of ER-positive breast cancer cells in vivo.^326^ Moreover, our recent study showed that MLN4924 activates PKM2 via inducing its tetramerization to promote glycolysis as a cellular protective mechanism in response to MLN4924 cytotoxicity. The combination of MLN4924 with PKM2 inhibitor shikonin synergistically inhibits growth of breast cancer cells in both in vitro cell culture setting and in vivo xenograft model.^237^

MLN4924 also act as a radio-sensitizer to sensitize a variety of cancer cells to radiotherapy. In pancreatic cancer cells, MLN4924 selectively sensitizes cancer cells, but not normal cells to radiation therapy by increasing radiation-induced DNA damage and apoptosis via causing the accumulation of CRL substrates, CDT1 and WEE1.^370^ Knockdown of CDT1 and WEE1 partially rescues MLN4924-induced enhancement of radiation effects, suggesting their causal roles.^370^ Likewise, MLN4924 also synergizes head and neck squamous carcinoma cells to irradiation in both in vitro cell culture and in vivo nude mice models, which is mainly attributed to the stabilization of CDT1 and induction of replication.^418^ In breast cancer cells, MLN4924 confers radio-sensitization through a p21-dependent mechanism, which is partially rescued by p21 knockdown.^419^ Similarly, MLN4924 serves as an effective radio-sensitizer by significantly enhancing irradiation-induced cell-cycle arrest and apoptosis in hormone refractory prostate cancer, through a mechanism of WEE1/p21/p27 accumulation.^420^ Finally, the activity of CRL1^FBXW7^ ligase is required for NHEJ repair in response to ionizing radiation via inducing XRCC4 polyubiquitylation via the K63 linkage. Thus, MLN4924 inactivation of CRL1^FBXW7^ in human cancers could be a potent strategy for increasing the efficacy of radiotherapy.^153^

Targeting immune checkpoints by blockage of programmed cell death protein 1 (PD-1) and its ligand PD-L1 have been approved by FDA for the treatment of several human cancers.^421^ The abundance of PD-L1 levels on tumor tissues often predicts the efficacy of PD-L1 inhibitors.^422^ Notably, the abundance of PD-L1 is regulated by ubiquitin ligases, such as CRL1 ^β-TrCP^ and CUL3^SPOP^, via proteasome-degradation pathway.^423,424^ MLN4924 upregulates PD-L1 in cancer cells via directly blocking the PD-L1 degradation by CRL E3,^423^ or indirectly by inactivating CRL1^FBXW7^ to cause accumulation of c-MYC, which transactivates PD-L1 expression.^408^ Thus, MLN4924 enhances the efficacy of anti-PD-L1 therapy through PD-L1 induction, and exerts the synergistic tumor suppressive effect in vivo.^408,425^ The deficient mismatch repair (dMMR) phenotype, also known as microsatellite instability (MSI), is a significant feature of certain types of human cancers, which is associated with chemotherapy resistance.^426–428^ A recent study showed that dMMR-induced destabilizing mutations triggers proteome instability in dMMR tumors, causing the accumulation of large number of misfolded proteins. The dMMR cells, therefore, develop a NEDD8-dependent degradation system to clean these misfolded proteins.^429^ As a result, blocking the NEDD8-dependent clearance pathway by MLN4924 increases the accumulation of misfolded protein aggregates with severe immunogenic cell death. The combination of MLN4924 and PD-1 inhibition, therefore, leverages this immunogenic cell death, with synergistically improving the efficacy over either treatment alone.^429^ Finally, MLN4924 was found to sensitize human cancer cells to VSVΔ51 oncolytic virotherapy via blocking the type 1 interferon (IFN-1) response through repression of interferon-stimulated growth factor 3 (ISGF3) in a neddylation-dependent manner, and NF-κB nuclear translocation.^430^ Taken together, MLN4924 could serve as a potential sensitizer when in combination with chemo, radio or immune-therapy in clinical application.

### Other NAE inhibitors

The success of MLN4924 sparked a surge in the discovery of additional NAE inhibitors, and various classes of NAE inhibitors has then been reported or under investigation, including covalent NAE inhibitors, non-covalent NAE inhibitors and even NAE agonizts.^345^ TAS4464, with structural similarity to MLN4924, is another potent and selective covalent NAE inhibitor with an impressive IC50 value of 0.96 nM.^431^ Few preclinical studies have shown that TAS4464 has potent growth-inhibitory effects against various cancer cell lines, derived from colorectal cancer, endometrial carcinoma, leukemia, liver cancer, ovarian cancer, pancreatic cancer and renal cell carcinoma in both in vitro and in vivo models.^431–434^ Mechanistically, TAS4464 induces sub-G1 arrest and apoptosis in cancer cells.^433,434^ Biochemically, TAS4464 blocks cullin neddylation and causes accumulation of CRL substrates, leading to the activation of extrinsic and intrinsic apoptotic pathways.^431^ Like MLN4924, TAS4464 also inactivate NF-κB pathways in cancer cells,^433^ thereby interfering with the signaling pathways associated with inflammation and cell survival. However, TAS4464 was advanced to Phase I clinical trial, but was terminated due to the liver toxicity.^432^

In addition to covalent inhibitors like MLN4924 and TAS4464, there are also few non-covalent NAE inhibitors being investigated. These inhibitors target the activity of NAE through non-covalent binding and have shown potential in suppressing tumor growth in vitro and in vivo,^435–445^ although none of them has been advanced to the clinical trials. Interestingly, a NAE agonist, first reported in 2020, showed promising antiproliferative activity against various cancer cell lines.^446^ In contrast to NAE inhibitors, this NAE agonist activated neddylation and induced morphology changes, apoptosis, and cell cycle arrest in cancer cells. It also suppresses tumor growth in xenograft tumor models. Thus, it appears that cancer cells are susceptible to alterations in neddylation pathway, whose inhibition or activation could affect the growth of cancer cells. NAE agonist might be a potential direction for future investigation in NAE-targeted drugs for cancer therapy.

Although MLN4924 and TAS4464 achieved many successes in preclinical studies, their blockage of the entire neddylation pathway bears unavoidable intrinsic toxicity to normal cells. In fact, in clinical trials, MLN4924 has shown a limited overall response rate (17%) in patients with AML or MDS (ClinicalTrials.gov Identifier: NCT00911066), and one of TAS4464 clinical trials was terminated due to liver toxicity (ClinicalTrials.gov Identifier: NCT02978235). Current efforts in the discovery of neddylation pathway inhibitors have focused on neddylation enzymes downstream of E1.

### Targeting E1–E2 interaction

The pivotal role of E2 conjugating enzymes in the NEDD8 conjugation process is essential, as they are responsible for engaging specific E3 ligases. Positioned centrally, E2 forms a vital connection, linking with both E1 and E3 enzymes, thus maintaining a crucial bridge between them.^324^ Targeting E2s, therefore, can be done by disrupting the catalytic core of E2 enzymes, or by blocking the interaction between E1 and E2, or E2 and E3. The crystal structures of UBE2M or UBE2F in complex with UBA3 have been resolved,^11,42,96^ providing the base for structure-based drug design and virtual screen. However, it’s still challenging due to the fast conformational changes and the absence of deep druggable pockets on its catalytic sites.^447^ Our group recently identified few potential pockets in UBE2F responsible for the UBA3-UBE2F interactions, and conducted a virtual screening which led to the discovery of a small molecule inhibitor HA-1141.^448^ HA-1141 directly binds to UBA3 in vitro and in vivo and effectively block neddylations of CUL 1-5, suggesting it targets E1. Unexpectedly, HA-1141 also induces non-canonical endoplasmic reticulum (ER) stress and PKR-mediated terminal integrated stress response (ISR), leading to activated ATF4, decreased protein synthesis and inhibited mTORC1 activity. HA-1141 showed a sound inhibitory effect in both cultured lung cancer cells and an in vivo lung tumor xenograft model.^448^

The same virtual screening also identified a small molecule HA-9104, which selectively targets the UBE2F-CRL5 axis.^449^ Specifically, HA-9104 binds to UBE2F, and blocks CUL5 neddylation to cause accumulation of CRL5 substrate NOXA, resulting in induction of apoptosis in lung cancer cells both in vitro cell culture setting and in vivo xenograft model.^449^ Medicinal chemistry-based SAR (Structure and Activity Relationship) study is under the way to optimize anticancer activity of these compounds.

In another recent study, Mamun et al. established a HTRF based drug screen focusing on searching for UBE2M inhibitors.^450^ The screen and subsequent biochemical and cellular assays discovered Micafungin, an antifungal agent, as a neddylation inhibitor targeting UBE2M. Micafungin interacts with UBE2M via occupying the catalytic cysteine (Cys111) in UBE2M, thus inhibiting neddylation to accumulate CRLs substrates, resulting in suppression of survival and migration of gastric cancer cells.^450^

### Targeting DCN1-UBE2M

DCN1 (defective in cullin neddylation 1) is a co-E3 ligase of RBX1 that forms a complex with UBE2M to enhance neddylation of cullins.^64^ Importantly, the crystal structure analysis revealed that DCN1–UBE2M interaction is amenable for the design of small-molecule inhibitors.^96,451^ To this end, a piperidine-based chemical probe, designated as NAcM-HIT, was discovered through a time-resolved fluorescence energy transfer (TR-FRET) based ligand competition assay from a library of 600,000 chemicals. NAcM-HIT specifically disrupts the DCN1-UBE2M binding, and inhibits the DCN1-dependent neddylation reaction.^451^ After a series of optimization, a NAcM-HIT analog was synthesized, which has 100-fold increase in potency, but still with low stability.^452^ Through a series of rational structure-based design and empirical chemistry approaches, a more potent and stable compound, NAcM-OPT was identified, which selectively disrupts the DCN1-UBE2M interaction and inhibit neddylation of CUL1 and CUL3, without affecting neddylation of CUL2, CUL4 and CUL5.^453^ Subsequent studies from the same group disclosed another series of pyrazolo-pyridones inhibitors, represented by compound **2**^454^ and compound **40,**^455^ with improved efficacy and reasonable oral bioavailability.

Simultaneously, the Wang group, through a structure-based virtual screen, identified the high-affinity, cell-permeable, drug-like peptidomimetic small-molecules, DI-591 and DI-404, which selectively inhibit the DCN1-UBE2M interaction.^456,457^ Unlike NAcM-OPT and MLN4924, DI-591/DI-404 specifically blocks CUL-3 neddylation without affecting, if any, the neddylation of other cullins. As a result, DI-591/DI-404 cause the accumulation of NRF2, a CUL-3 substrate, in a dose-dependent manner in multiple cancer cell lines.^456^ However, DI-591/DI-404 fail to show any cytotoxicity in both human cancer cells at concentrations up to 20 µM, suggesting the dispensable functions of CRL3 activity in determining cell survival. Based upon DI-591, the Wang group further designed a class of covalent DCN1 inhibitors and discovered two potent, selective and covalent DCN1 inhibitors, designated as DI-1548 and DI-1859.^458^ Both inhibitors inhibit CUL-3 neddylation in cancer cells at low nanomolar concentrations with 2–3 orders of magnitude more potent than DI-591. Impressively, DI-1859 induces a robust increase of NRF2 protein in mouse liver and effectively protects mice from liver toxicity induced by acetaminophen. Given that activation of NRF2 has been pursued as a promising therapeutic approach for treatment of multiple sclerosis,^459^ DI-1859 could be a potential therapeutic agent for the treatment of this disease. DI-1859 may also have a potential therapeutic application to protect normal tissues from acute toxicity induced by oxidative stress through upregulation of NRF2 via blocking CRL3 activity.^460^

Toward the DCN-1 target, the Liu and Zhao group also discovered several small molecule inhibitors with distinct chemical structures. Triazolo[1,5-α] pyrimidine-based inhibitor WS-291 and its successor WS-383 showed inhibitory effect on neddylation of CUL-1 and CUL-3 in gastric cancer cell lines.^461^ Another pyrimidines-based small molecular inhibitor DC-2 could specifically inhibits CUL-3 neddylation with IC_50_ around 15 nM,^462^ and further SAR yielded DN-2 with even more potent efficacy (IC_50_ = 9.6 nM).^463^ The Liu group also reported a phenyltriazole thiol-based inhibitor SK-464 with IC_50_ value of 26 nM, bringing a new class of potent DCN1 inhibitors into this field.^464^ Although none of the reported DCN1/UBE2M inhibitors has entered a clinical trial, they provided guidance for future drug design and discovery on this target.

### Targeting neddylation E3s

From a broader perspective, achieving therapeutic targeting of RING E3s in neddylation holds the promise of greater selectivity and fewer side effects. However, no such specific inhibitors targeting RBX1 or RBX2/SAG has been identified. From a structural perspective, RBX1 or SAG lacks a well-defined catalytic pocket, making it challenging to identify or design small-molecule inhibitors.^465^ In addition, the ubiquitylation and neddylation processes both involve a dynamic enzymatic cascade, featuring numerous transient and temporary protein-protein interactions, which makes the RING E3s difficult to target.^465^ Despite these challenges, several research teams have put the efforts to discover small molecule inhibitors for this seemingly “undruggable” target. Indeed, one reference compound, designated as C64, was extensively characterized for selective binding at the RBX1-binding grooves with VLYRLWLN motif on CUL1-7,^466^ through various approaches, including a series of bioinformatics analysis on the binding patterns between RBX1 and seven cullins, molecular dynamics (MD) simulations to define critical binding sites and to predict key conformations influencing RBX1-Cullin binding stability, and structure-based virtual screening and docking analyses. However, the study did not confirm the inhibitory activity of these small molecules, although the analysis of the RBX1-Cullin binding site and conformation could provide valuable insights for designing small-molecule inhibitors targeting RBX1-induced cullin neddylation.

Our group recently developed an Alpha-Screen-based high throughput screen (HTS) assay, and identified a natural compound derived from cotton seed named gossypol that can effectively block neddylation of CUL1 and CUL5 by directly binding to RBX1-CUL1 or SAG-CUL5 complex, respectively.^467^ In this way, gossypol inhibits cullins neddylation in multiple cancer cell lines but selectively causes accumulation of the CRL1 substrate MCL1, and CRL5 substrate NOXA.^467^ By inducing the accumulation of NOXA, gossypol induces apoptosis in few cancer cell lines. Given that MCL1 is a pro-survival Bcl-2-like protein, and gossypol-induced MCL1 links to the less efficacy of gossypol, we found combination of gossypol with MCL1 inhibitor S63845 indeed achieve a better growth inhibition of cancer cells.^467^ However, the underlying mechanism for gossypol to selectively cause accumulation of MCL1 and NOXA, but not other substrates of CRL1 and CRL5, is a subject for future investigation.

## Conclusion and future perspectives

**Fig. 8:**
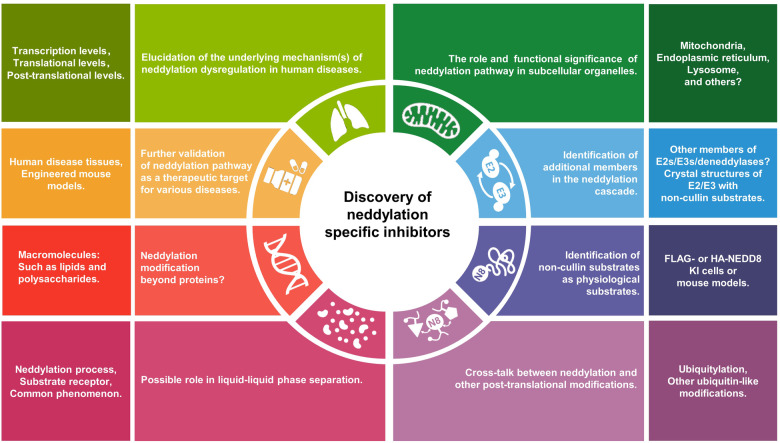
Future perspectives. A total of nine research directions are proposed for future investigations, covering from basic mechanistic study, target identification and validation, the use of animal disease models to eventually discover specific neddylation inhibitors for clinical trials to treat various human diseases with abnormal activation of the neddylation pathway (see text for details). Created at BioRender.com

In this review, we describe how neddylation pathway modulates the biochemical activities of cullin and non-cullin substrates by catalyzing the NEDD8 attachment, leading to activation of CRLs or altered activities, stability or subcellular localization of a variety of non-cullin substrates. We also summarize the essential role of neddylation pathway in the maintenance of healthy cellular homeostasis, and its active involvement, upon abnormal activation or dysregulation, in the development of various human diseases. Finally, we discuss current efforts in the discovery and development of small molecular inhibitors of neddylation pathway and its potential application in the treatment of human diseases, mainly human cancer. However, there are quite a few unsolved puzzles and open areas in the field of neddylation research. Below, we list a number of possible directions for future investigations (Fig. 8).

### What is the underlying mechanism(s) of neddyltion dysregulation in human diseases?

The exact underlying mechanisms by which NEDD8 and neddylation enzymes are altered and in the most cases over-activated in human diseases, such as cancer, remains elusive. The future studies on this aspect should be directed to a systematic elucidation of possible alterations at 1) the transcription levels, including epigenetic modifications of gene promoters and enhancers; 2) the mRNA translational levels including RNA modifications, such as N^6^-methyladenosine (m^6^A), 5-methylcytosine (m^5^C), and 7-methylguanosine (m^7^G);^468^ 3) the post-translational levels, including phosphorylation, acetylation, methylation, even ubiquitylation, among many others that affect their stability^469^ as well as the regulators that affect their activities, such as inositol hexakisphosphate (IP6) and other metabolites.^470^

### How many reported non-cullin substrates are physiological substrates?

While cullin family members are well-established as the physiological substrates of neddylation, almost all non-cullin substrates were identified and characterized in the cell culture settings with overexpression or knockdown approaches, which raises a serious consideration as to whether they are indeed physiological substrates of neddylation.^17^ Furthermore, unlike cullins, none of non-cullin substrates showed in western blotting a fast-migrating (non-neddylated form) and a slow-migrating (neddylated form) bands with conversion of neddylated to non-neddylated form upon treatment of neddylation inhibitor (e.g., MLN4924) or knockdown of neddylation enzymes.^12^ The big challenge in the field is to validate (for reported non-cullin substrates) and identify novel physiological substrates of neddylation, if any. It is known that under the condition of NEDD8 overexpression, NEDD8 is mistakenly recognized as ubiquitin by UAE, an ubiquitin activating enzyme E1, and conjugated onto its ubiquitin substrates, leading to erroneous identification of neddylation substrates. The possible solution to this scenario is to generate cell lines expressing the CRISPR-Cas9-mediated knock-in of NEDD8 with an N-terminal FLAG- or HA-tag, followed by FLAG-/HA-based pull-down and affinity purification to identify true endogenous substrates. One step further is to generate a knock-in mouse model, in which FLAG- or HA-tagged Nedd8 is expressed in the physiological level to facilitate the identification of spatiotemporal-specific neddylation substrates. Moreover, the combination of deconjugation-resistant form of NEDD8 with a specific antibody against the unique NEDD8 remnant would offer additional options to identify new neddylation substrates.^56,471^

### Are there additional members in the neddylation cascade?

Although the biochemical process of neddylation modification is well established, few questions could be asked: 1) are there any additional E2s for neddylation, beyond UBE2M and UBE2F? 2) can UBE2M or UBE2F act as an E2 for ubiquitylation or other ubiquitin-like modifications? Indeed, we found that UBE2M can act as an ubiquitin E2 for CRL3 and Parkin E3 ubiquitin ligases^55^; 3) It will not be surprising that additional ubiquitin E3s will be identified to serve as neddylation E3s, and deubiquitylases as deneddylases. 4) While few crystal structures of neddylation E2/E3 complexed with cullin substrates have been resolved,^95–98,100^ no such structure was resolved/available for non-cullin substrates.

### Does the neddylation pathway function in subcellular organelles?

Although neddylation plays a regulatory role in the morphology and function of mitochondria,^109^ detailed underlying mechanism remains elusive. For examples, are neddylation enzymes or cullin substrates localized in the mitochondria and if so, in what compartment? And whether and how the neddylation-CRL system modifies mitochondrial proteins, thus affecting the process of ROS generation or energy production? Finally, are neddylation components localized in other subcellular organelles, such as endoplasmic reticulum or lysosome?

### Possible role in liquid–liquid phase separation

Liquid–liquid phase separation (LLPS) is ubiquitous phenomenon underlying the formation of membraneless organelles in eukaryotic cells to facilitate local biochemical reaction.^472^ Several studies have shown that neddylation modulates LLPS. For examples, 1) neddylation inhibitors MLN4924 and TAS4464 promote phase separation of PML/RARα;^473^ 2) DAXX drives LLPS of SPOP, a well-known receptor for CRL3, to promote DAXX ubiquitylation and degradation;^474^ 3) CRL5^ASB11^ promotes the ubiquitylation of PAICS, leading to the recruitment of UBAP2 to trigger LLPS to form the purinosome.^475^ The open questions include 1) does LLPS occurs during the neddylation process under specific physiological or pathological/stressed conditions? 2) whether and under what conditions that the substrate receptors of CRL drive their own LLPS to rapidly promote the ubiquitylation of substrates? 3) does LLPS regulation by the neddylation-CRL axis is a common phenomenon to facilitate ubiquitylation of substrates?

### Cross-talk between neddylation and other post-translational modifications

The cross-talk between neddylation and ubiquitylation is frequently observed with the same or opposite effect. For example, either neddylation or ubiquitylation of yeast CUL4 orthologue Rtt101 causes its catalytic activation.^48^ Similarly, in response to DNA damage both NEDD8 and ubiquitin accumulate at the DNA damage sites, and the poly-neddylation and poly-ubiquitylation of histones are functionally redundant.^44,476,477^ In contrast, neddylation or ubiquitylation of TGFβRII elicit distinct biochemical consequence. While ubiquitylation leads to degradation, neddylation mediates signal transduction through clathrin-mediated endocytosis.^478^ Furthermore, neddylation on a given protein often inhibits its ubiquitylation and subsequent degradation.^47,131,238,251,278,479^ An important open question is how a cell coordinates these two types of modifications to gain the growth advantage. Besides ubiquitylation, much less studies were conducted to elucidate the cross-talks between neddylation and other types of ubiquitin-like modifications, such as sumoylation.^480^ Thorough understanding these cross-talks will deepen our understanding of neddylation regulation of various biological processes.

### NEDD8 modifications of itself or other macromolecules

NEDD8 itself may undergo post-translational modifications, since numerous proteomic studies have shown the presence on NEDD8 of ubiquitylation, phosphorylation, acetylation, and succinylation sites.^481–484^ However, no follow-up studies, except phosphorylation,^485^ were conducted to confirm these possible modifications, not to mention their biochemical and functional significance of these modifications.

NEDD8 is also found to incorporate into an existing ubiquitin chain to form mixed NEDD8- and Ub-modified conjugates.^15,16,23^ It remains to determine whether this process is physiologically relevant or merely an experimental artifact? If the former is true, what is the biochemical consequence or biological significance for this mix conjugation on a protein?

Finally, few recent studies have shown that ubiquitylation can occur on few other macrobiomolecules such as glucosaccharides^486^ and lipopolysaccharide.^487^ It is completely unknown but very possible that these macromolecules are also subjected to neddylation modification. It will be an interesting subject for future investigation.

### Further validation of neddylation pathway as a therapeutic target for various diseases

The dysregulation of neddylation pathway frequently occurs in several human diseases, particularly in a variety of human cancers, as evidenced by overexpression of NEDD8 and neddylation enzymes.^12,21,72^ However, these association studies did not reveal any causal or consequential relationship between these alterations in the disease development. Thus, to pinpoint genetically the causal or consequent effects, engineered mouse models, including organ-specific deletion or transgenic expression, should be established alone or in combination with known mouse models for various diseases, including cancer (e.g., Kras activation,^488^ p53/pTen loss^489,490^), metabolic disorders (e.g., Leptin-deficient *ob/ob* model, leptin receptor-deficient *db/db* model^491^), neurodegenerative diseases (e.g., APP/PS1 model for Alzheimer’s disease, mutant α-Synuclein or mutant LRRK2 transgenic models for autosomal dominant Parkinson’s disease),^492^ immune disorders (e.g., *SAMP1/YitFc* (*Samp*) mice for Crohn’s disease,^493^ *K/BxN* mice for rheumatoid arthritis,^494^ and cardiovascular diseases (e.g., TAC mice^495^), among others.

### Discovery of neddylation-specific inhibitors

Although neddylation pathway has been validated as a promising anticancer target,^12,21^ only two inhibitors of neddylation E1 (MLN4924 and TAS4464) were advanced into the clinical trials,^354,432^ and none of them is granted by FDA for the treatment of human cancer, although preclinical studies showed impressive anti-cancer effect.^18,431^ The fact that E1 inhibitors block the entire neddylation pathway, the cytotoxicity is inevitable. Furthermore, MLN4924 bears a number of off-target effects.^496^ Thus, the further efforts should be more focused on the discovery and development of specific inhibitors targeting neddylation E2s, UBE2F or UBE2M or E3s, such as RBX1 or RBX2, leading to indirect inhibition of selective CRLs. Although several such inhibitors were reported,^448–458,497^ extensive studies with guidance of the crystal structures and AI simulation,^498^ are needed to discover much potent and selective inhibitors for the advancement to the clinical trials.
